# Pressure overload induces ISG15 to facilitate adverse ventricular remodeling and promote heart failure

**DOI:** 10.1172/JCI161453

**Published:** 2023-05-01

**Authors:** Veera Ganesh Yerra, Sri Nagarjun Batchu, Harmandeep Kaur, MD Golam Kabir, Youan Liu, Suzanne L. Advani, Duc Tin Tran, Shadi Sadeghian, Phelopater Sedrak, Filio Billia, Uros Kuzmanov, Anthony O. Gramolini, Deema O. Qasrawi, Evgeniy V. Petrotchenko, Christoph H. Borchers, Kim A. Connelly, Andrew Advani

**Affiliations:** 1 Keenan Research Centre for Biomedical Science and Li Ka Shing Knowledge Institute, St. Michael’s Hospital, Toronto, Ontario, Canada.; 2 Temerty Faculty of Medicine, University of Toronto, Toronto, Ontario, Canada.; 3 Toronto General Hospital Research Institute, University Health Network, Toronto, Ontario, Canada.; 4 Ted Rogers Centre for Heart Research, Toronto, Ontario, Canada.; 5 Department of Physiology, University of Toronto, Toronto, Ontario, Canada.; 6 Segal Cancer Proteomics Centre, Lady Davis Institute for Medical Research, Jewish General Hospital, McGill University, Montreal, Quebec, Canada.; 7 Gerald Bronfman Department of Oncology, McGill University, Montreal, Quebec, Canada.

**Keywords:** Cardiology, Heart failure

## Abstract

Inflammation promotes adverse ventricular remodeling, a common antecedent of heart failure. Here, we set out to determine how inflammatory cells affect cardiomyocytes in the remodeling heart. Pathogenic cardiac macrophages induced an IFN response in cardiomyocytes, characterized by upregulation of the ubiquitin-like protein IFN-stimulated gene 15 (ISG15), which posttranslationally modifies its targets through a process termed ISGylation. Cardiac ISG15 is controlled by type I IFN signaling, and ISG15 or ISGylation is upregulated in mice with transverse aortic constriction or infused with angiotensin II; rats with uninephrectomy and DOCA-salt, or pulmonary artery banding; cardiomyocytes exposed to IFNs or CD4^+^ T cell–conditioned medium; and ventricular tissue of humans with nonischemic cardiomyopathy. By nanoscale liquid chromatography–tandem mass spectrometry, we identified the myofibrillar protein filamin-C as an ISGylation target. ISG15 deficiency preserved cardiac function in mice with transverse aortic constriction and led to improved recovery of mouse hearts ex vivo. Metabolomics revealed that ISG15 regulates cardiac amino acid metabolism, whereas ISG15 deficiency prevented misfolded filamin-C accumulation and induced cardiomyocyte autophagy. In sum, ISG15 upregulation is a feature of pathological ventricular remodeling, and protein ISGylation is an inflammation-induced posttranslational modification that may contribute to heart failure development by altering cardiomyocyte protein turnover.

## Graphical abstract

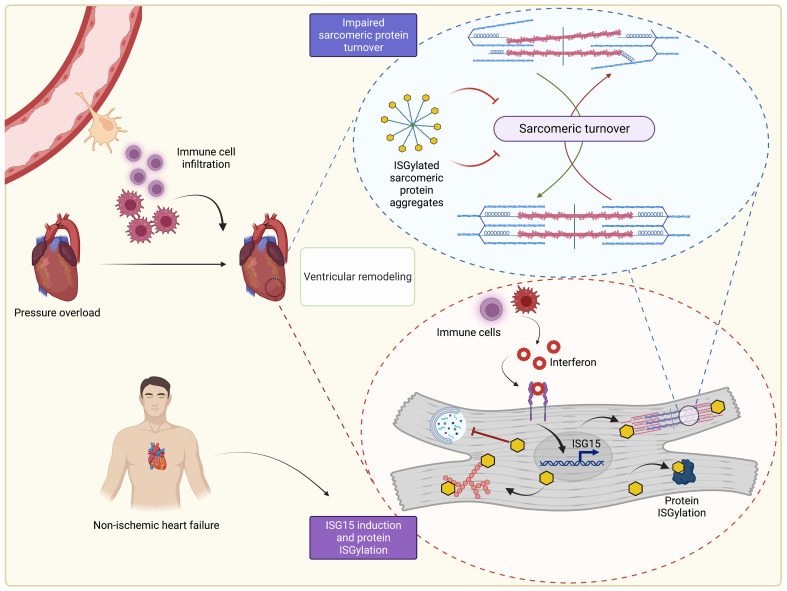

## Introduction

Heart failure is the clinical manifestation of a heterogeneous group of often overlapping conditions that impair the effective emptying or filling of the cardiac ventricles. Despite the heterogeneity of its etiology, heart failure is often preceded by a period of ventricular remodeling, a pathological process involving the abnormal change in size, shape, function, or cellular composition of the cardiac chambers in response to load or injury (1). This change in cellular composition includes the accumulation of several different immune cell subpopulations that, through their proinflammatory actions, contribute to adverse remodeling and cardiac dysfunction. For instance, ischemic cell death after myocardial infarction can trigger an IFN response that impairs left ventricular (LV) function and limits survival (2). Immune cell recruitment and pathological remodeling are also key events during the pathogenesis of nonischemic cardiomyopathy, for example that caused by pressure overload (3, 4), a major contributor to heart failure development. However, the role of the IFN response in ventricular remodeling caused by pressure overload has not previously been established.

Among the different immune cell subpopulations that accumulate in the myocardium in response to pathological hemodynamic stress, CCR2^+^ monocyte-derived macrophages have emerged as central players in adverse ventricular remodeling and the development of heart failure (5–9). In addition to their roles in antigen presentation and phagocytosis, macrophages secrete a range of bioactive molecules, including inflammatory chemokines and cytokines, potentially including IFNs (10, 11). In cardiomyocytes, activation of IFN receptors, Toll-like receptors, and cytosolic pattern recognition receptors can, in turn, induce an IFN response that is characterized by the induction of dozens of IFN-stimulated genes (ISGs) (12–14). For instance, one of the most highly inducible ISGs, ISG15 ubiquitin like modifier (IFN-stimulated gene of 15 kDa; ISG15), is expressed by cardiomyocytes in response to the type I IFN IFN-β, and it is induced in cardiomyocytes in response to viral infection (15, 16). One of the primary functions of ISG15 is to covalently conjugate to lysine residues of actively translated proteins through a process termed ISGylation (17). The importance of ISGylation as a posttranslational modification has been appreciated by the virology community for years (18). However, ISGylation has also garnered particular interest recently because one mechanism by which SARS-CoV-2 (the virus responsible for COVID-19) evades the host innate immune response is through its papain-like protease, which cleaves ISG15 from viral proteins (19). In the heart, cardiomyocyte ISG15 posttranslationally modifies coxsackievirus B3 viral protein and attenuates myocarditis (15). Viral proteins, though, are not the only proteins to be ISGylated. To date, several hundred cellular ISG15 protein substrates have been identified, resulting in context-specific gain or loss of protein function (12, 20). However, whether ISG15 induction and protein ISGylation take place during the sterile inflammation associated with pressure overload–induced ventricular remodeling and, if so, whether this has any effect on heart function itself have not been defined.

In this study, our goal was to understand how accumulating immune cells can affect ventricular remodeling and heart function in response to pressure overload. We found that cardiac immune cells induce an IFN response in cardiomyocytes that is characterized by ISG15 upregulation. Through exploring the effects of ISG15 in cardiomyocytes, we define protein ISGylation as an inflammation-induced posttranslational modification important in the development of pathological ventricular remodeling and cardiac dysfunction.

## Results

### Recruited cardiac immune cells induce an IFN response in cardiomyocytes that is characterized by ISG15 upregulation.

**Figure 1:**
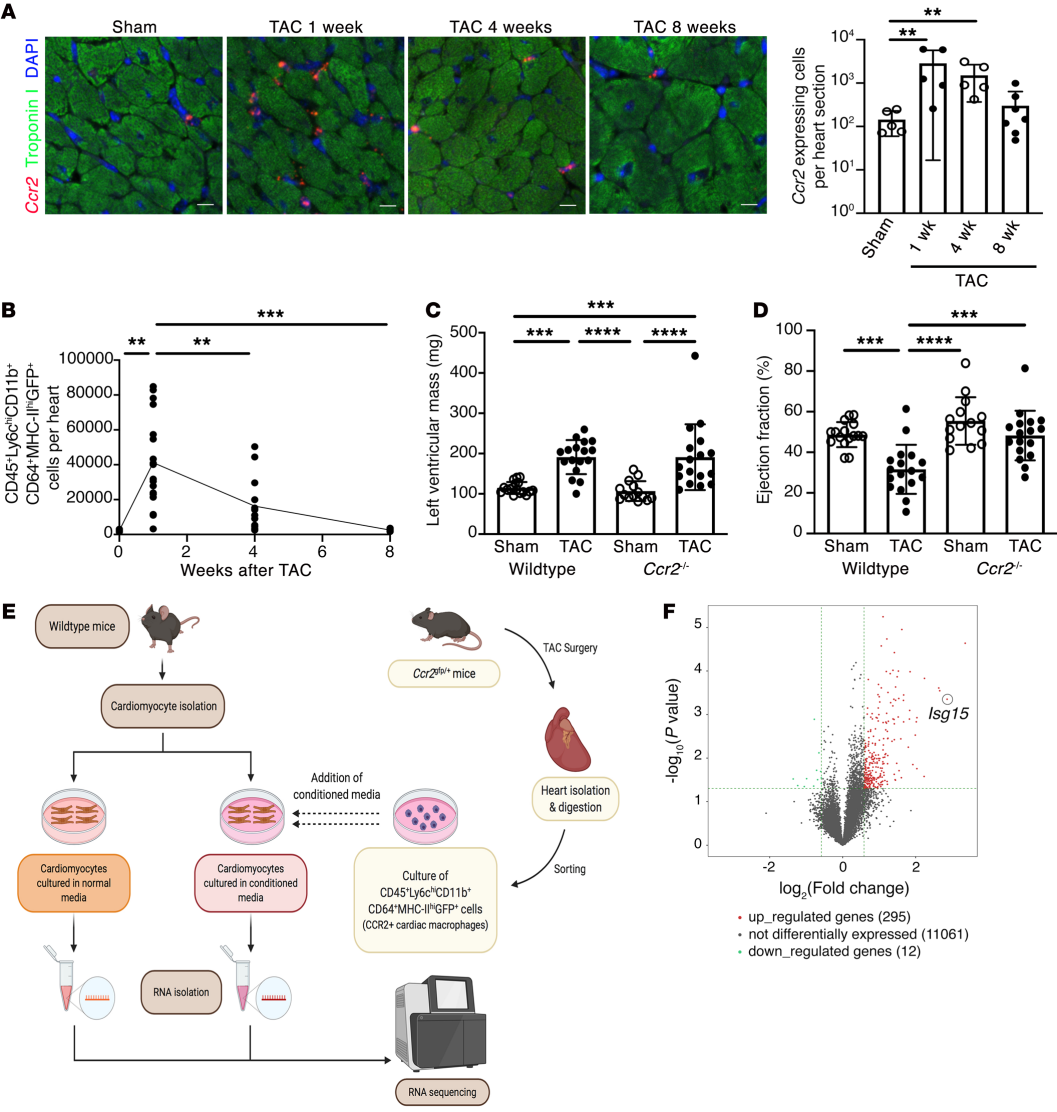
CCR2^+^ macrophage accumulation impairs cardiac function in pressure overload. (**A**) RNAscope in situ hybridization for *Ccr2* and immunostaining for troponin I in mouse hearts 8 weeks after sham surgery or 1, 4, or 8 weeks after transverse aortic constriction (TAC). Sham, *n* = 5; 1 week TAC, *n* = 5; 4 weeks TAC, *n* = 5; 8 weeks TAC, *n* = 7. Scale bars: 10 μm. (**B**) Enumeration of CCR2^+^ cardiac monocyte-derived macrophages (CD45^+^Ly6C^hi^CD11b^+^CD64^+^MHC-II^hi^GFP^+^ cells) in *Ccr2^gfp/+^* mice. Control, *n* = 5; 1 week TAC, *n* = 18; 4 weeks TAC, *n* = 13; 8 weeks TAC, *n* = 6. (**C** and **D**) LV mass (**C**) and ejection fraction (**D**) in WT and *Ccr2^–/–^* mice 8 weeks after sham or TAC. WT sham, *n* = 16; WT TAC, *n* = 17; *Ccr2^–/–^* sham, *n* = 14; *Ccr2^–/–^* TAC, *n* = 17. (**E**) Design of RNA sequencing experiments. (**F**) Volcano plot of genes expressed by mouse cardiomyocytes exposed to CCR2^+^ cardiac macrophage–conditioned medium (*n* = 4 per condition). Values are mean ± SD. **P < 0.01, ***P < 0.001, ****P < 0.0001 by 1-way ANOVA followed by Dunnett’s post hoc test (**A**) or Tukey’s post hoc test (**B**–**D**).

**Figure 2:**
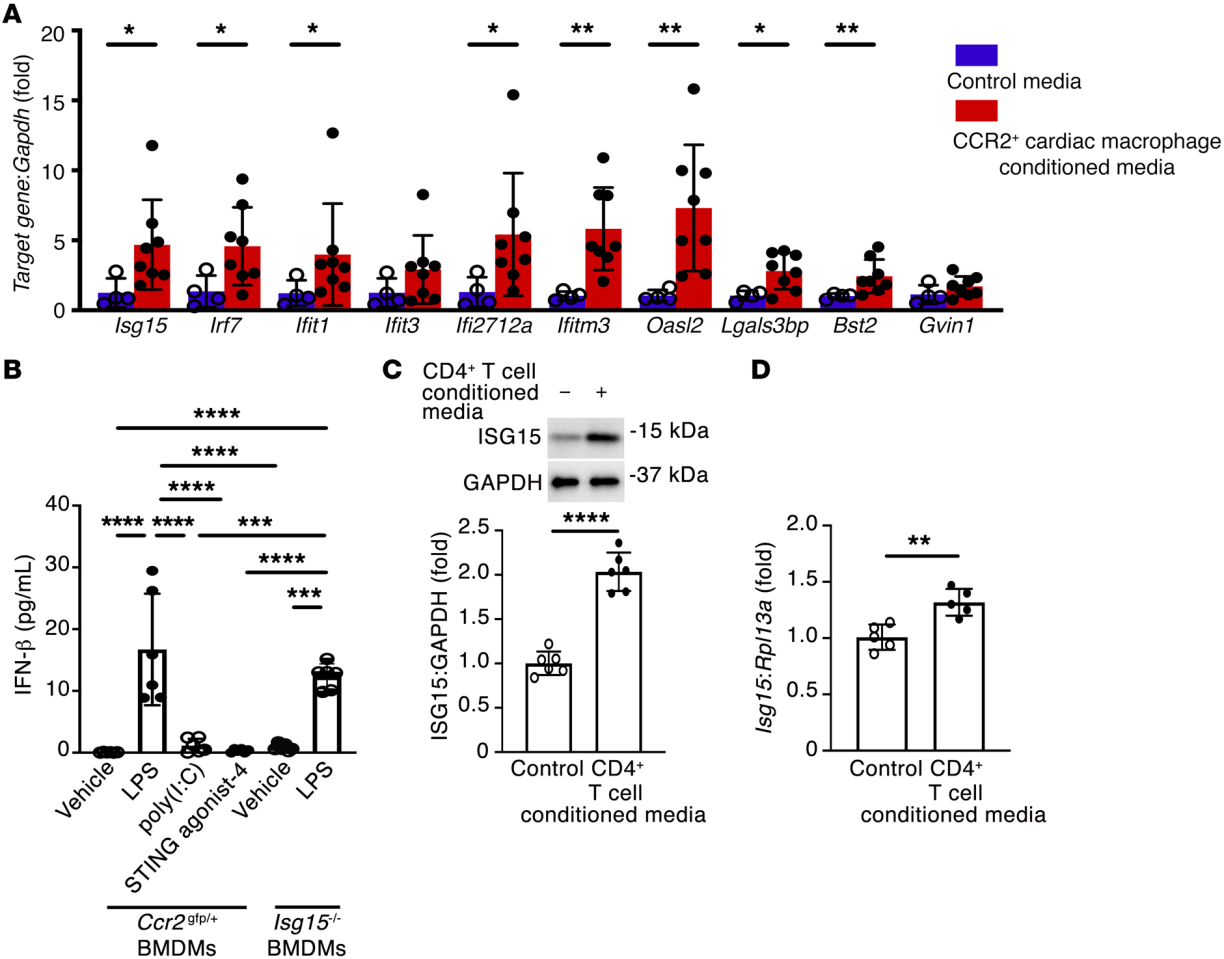
CCR2^+^ cardiac macrophages induce a cardiomyocyte IFN response. (**A**) qRT-PCR for IFN response genes (*Isg15*, *Irf7*, *Ifit1*, *Ifit3*, *Ifi2712a*, *Ifitm3*, *Oasl2*, *Lgals3bp*, *Bst2*, *Gvin1*) in mouse cardiomyocytes in medium conditioned by CCR2^+^ cardiac macrophages isolated from *Ccr2^gfp/+^* mouse hearts 1 week after TAC, or under control conditions. Control, *n* = 4; CCR2^+^ cardiac macrophage–conditioned medium, *n* = 8. (**B**) Culture medium IFN-β concentration in bone marrow–derived macrophages (BMDMs) from *Ccr2^gfp/+^* mice or *Isg15^–/–^* mice incubated with LPS (1 μg/mL), poly(I:C) (500 ng/mL), or STING agonist-4 (5 μmol/L) for 24 hours (*n* = 6 per condition). (**C** and **D**) Immunoblotting (**C**; *n* = 6 per condition) and qRT-PCR (**D**; *n* = 5 per condition) for ISG15 in mouse cardiomyocytes exposed to CD4^+^ T cell–conditioned medium for 24 hours. Values are mean ± SD. *P < 0.05, **P < 0.01, ***P < 0.001, ****P < 0.0001 by unpaired 2-tailed Mann-Whitney test (**A**), 1-way ANOVA followed by Tukey’s post hoc test (**B**), or unpaired 2-tailed Student’s *t* test (**C** and **D**).

In our first set of experiments, we sought to determine how immune cells recruited to the pressure-overloaded heart may affect cardiomyocytes. We began by studying CCR2^+^ macrophages because of their previously described contributions to adverse ventricular remodeling and heart failure development in mice (7) and humans (5). Using in situ hybridization (Figure 1A) and flow cytometry (Figure 1B and Supplemental Figure 1; supplemental material available online with this article; https://doi.org/10.1172/JCI161453DS1), we observed that CCR2^+^ macrophages accumulated in mouse hearts early (within 1 week) after transverse aortic constriction (TAC), gradually declining over an 8-week period. We subjected wild-type (WT) and *Ccr2^–/–^* mice (21) to sham or TAC surgery and followed the animals for 8 weeks (Supplemental Figure 2 and Supplemental Tables 1–3). Mean LV mass was increased equivalently in WT and *Ccr2^–/–^* TAC mice (Figure 1C), whereas ejection fraction (Figure 1D), fractional shortening, cardiac output, and stroke volume (Supplemental Figure 3) were all reduced in WT TAC mice and comparatively preserved in *Ccr2^–/–^* TAC mice, confirming a pathogenic role for CCR2^+^ macrophages in ventricular remodeling induced by pressure overload. CCR2^+^ cardiac macrophages were then isolated from *Ccr2^gfp/+^* mice 4 weeks after TAC, and the culture medium conditioned by these cells was applied to cardiomyocytes isolated from adult WT mice, before RNA isolation from the cardiomyocytes and RNA sequencing (Figure 1E). Among the 11,368 genes detected in adult mouse cardiomyocytes (fragments per kilobase of transcript per million [FPKM] ≥ 0.5 mean in 1 group), 295 (2.6%) were upregulated in cardiomyocytes cultured in medium conditioned by CCR2^+^ cardiac macrophages, and 12 (0.1%) were downregulated (Figure 1F) (fold change ≥ 1.5, *P* < 0.05). Hierarchical clustering of genes that were upregulated ≥2.5-fold (*P* < 0.05) revealed that dozens of these genes are involved in the IFN response (Supplemental Figure 4A). Gene Ontology and Kyoto Encyclopedia of Genes and Genomes (KEGG) analyses similarly identified upregulated biological processes and pathways linked with immune responses (Supplemental Tables 4 and 5), including NOD-like receptor signaling (*P* = 6.38 × 10^–15^, false discovery rate [FDR] 6.86 × 10^–13^) and viral myocarditis (*P* = 2.61 × 10^–8^, FDR 1.40 × 10^–6^) (Supplemental Table 5). We entered the gene symbol list from the heatmap (Supplemental Figure 4A) into the Interferome (v2.01) database (22) and observed that of the 42 differentially expressed genes, 39 had been described as being IFN response genes in mice (fold change ≥ 2.0) (Supplemental Figure 4B). Next, to confirm that CCR2^+^ cardiac macrophages induce an IFN response in mouse cardiomyocytes, we exposed cardiomyocytes to medium conditioned by CCR2^+^ cardiac macrophages isolated from mouse hearts 1 week after TAC before performing quantitative reverse transcription PCR (qRT-PCR). Of 10 selected ISGs, 8 were also increased by CCR2^+^ cardiac macrophage–conditioned medium at this earlier time point (*Isg15*, *Irf7*, *Ifit1*, *Ifi2712a*, *Ifitm3*, *Oasl2*, *Lgals3bp*, *Bst2*) (Figure 2A). Similarly, when we performed qRT-PCR of mRNA isolated from mouse hearts 1, 4, or 8 weeks after TAC surgery, we also observed an increase in transcript abundance of ISGs (*Isg15*, *Ifit1*, *Ifi2712a*, *Ifitm3*, *Oasl2*, *Bst2*, and *Gvin1*) (Supplemental Figure 5).

Having detected evidence of IFN pathway activation and ISG induction in cardiomyocytes exposed to CCR2^+^ cardiac macrophage–conditioned medium and in TAC hearts, we selected ISG15 for further study. We did this because (a) *Isg15* was upregulated more than 7-fold in our RNA sequencing experiment (*P* < 0.001) (Figure 1F); (b) *Isg15* mRNA levels were confirmed by qRT-PCR to be upregulated in cardiomyocytes exposed to CCR2^+^ cardiac macrophage–conditioned medium (Figure 2A); and (c) *Isg15* mRNA levels were increased in TAC hearts (Supplemental Figure 5). In examining the stimuli for ISG15 induction, we first measured concentrations of IFN-α and IFN-β in medium conditioned by CCR2^+^ cardiac macrophages. By multiplex assay (Supplemental Table 6), the median (range) concentration of IFN-β was 3.28 (0–6.54) pg/mL per 1,000 cells, whereas IFN-α concentrations were below the level of detection using high-sensitivity ELISA (assay range 2.38–152 pg/mL). To explore both the stimuli for macrophage IFN-β secretion and the effects of ISG15 itself on macrophage activation, bone marrow–derived macrophages (BMDMs) were isolated from either *Ccr2^gfp/+^* mice or *Isg15^–/–^* mice, first confirming by flow cytometry that BMDMs express CCR2 on their cell surfaces (Supplemental Figure 6). Activation of BMDMs with the Toll-like receptor 4 (TLR4) ligand lipopolysaccharide (LPS) induced IFN-β secretion into the culture medium, whereas the synthetic dsRNA polymer poly(I:C) or the cytosolic DNA sensing pathway activator STING agonist-4 did not (Figure 2B). Culture medium IFN-β concentration after LPS activation was similar in *Isg15^–/–^* BMDMs and *Ccr2^gfp/+^* BMDMs (Figure 2B). Lastly, we recognized that CCR2^+^ cardiac macrophages are not the only immune cells to be implicated in ventricular remodeling; for instance, CD4^+^ T cells also play an important role in the transition from cardiac hypertrophy to heart failure (23). Accordingly, we exposed cardiomyocytes to medium conditioned by splenic CD4^+^ T cells (Supplemental Figure 7), and we similarly observed an upregulation in ISG15 expression (Figure 2, C and D). Thus, the sterile inflammation that accompanies ventricular remodeling induces upregulation of the ubiquitin-like protein (Ubl) ISG15 in cardiomyocytes.

### Pressure overload induces ISG15 expression through type I IFN receptor signaling.

**Figure 3:**
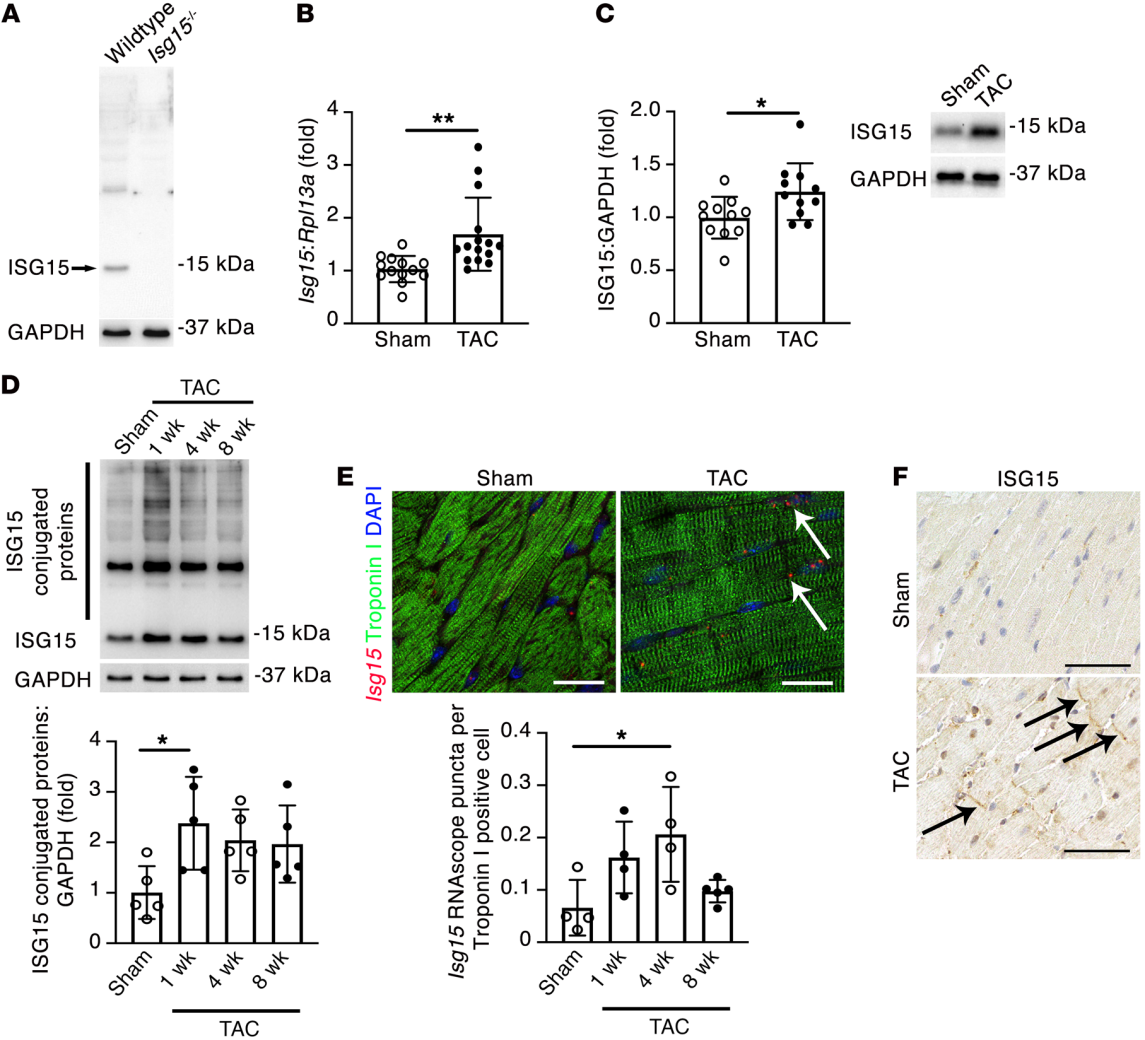
Cardiomyocyte ISG15 is upregulated in mouse hearts after TAC. (**A**) Immunoblotting of WT and *Isg15^–/–^* mouse hearts confirming specificity of the ISG15 antibody (clone 1H9L21). (**B**) qRT-PCR for *Isg15* in mouse hearts 8 weeks after sham or TAC. Sham, *n* = 13; TAC, *n* = 15. (**C**) Immunoblotting for ISG15 in mouse hearts 8 weeks after sham or TAC. Sham, *n* = 11; TAC, *n* = 11. (**D**) Immunoblotting for ISG15-conjugated proteins in mouse hearts 8 weeks after sham surgery or 1, 4, or 8 weeks after TAC (*n* = 5 per group). (**E**) RNAscope in situ hybridization for *Isg15* and immunofluorescence for troponin I in heart sections from mice 4 weeks after sham or TAC. The arrows mark *Isg15* RNAscope puncta in troponin I^+^ cardiomyocytes. Scale bars: 20 μm. *n* = 4 per group, except 8 weeks after TAC (*n* = 5). (**F**) Immunohistochemistry for ISG15 in mouse hearts 4 weeks after sham or TAC. The arrows mark positive immunostaining at, or close to, intercalated discs. Scale bars: 50 μm. Values are mean ± SD. *P < 0.05, **P < 0.01 by unpaired 2-tailed Student’s *t* test (**B** and **C**), or 1-way ANOVA followed by Dunnett’s post hoc test (**D** and **E**).

**Figure 4:**
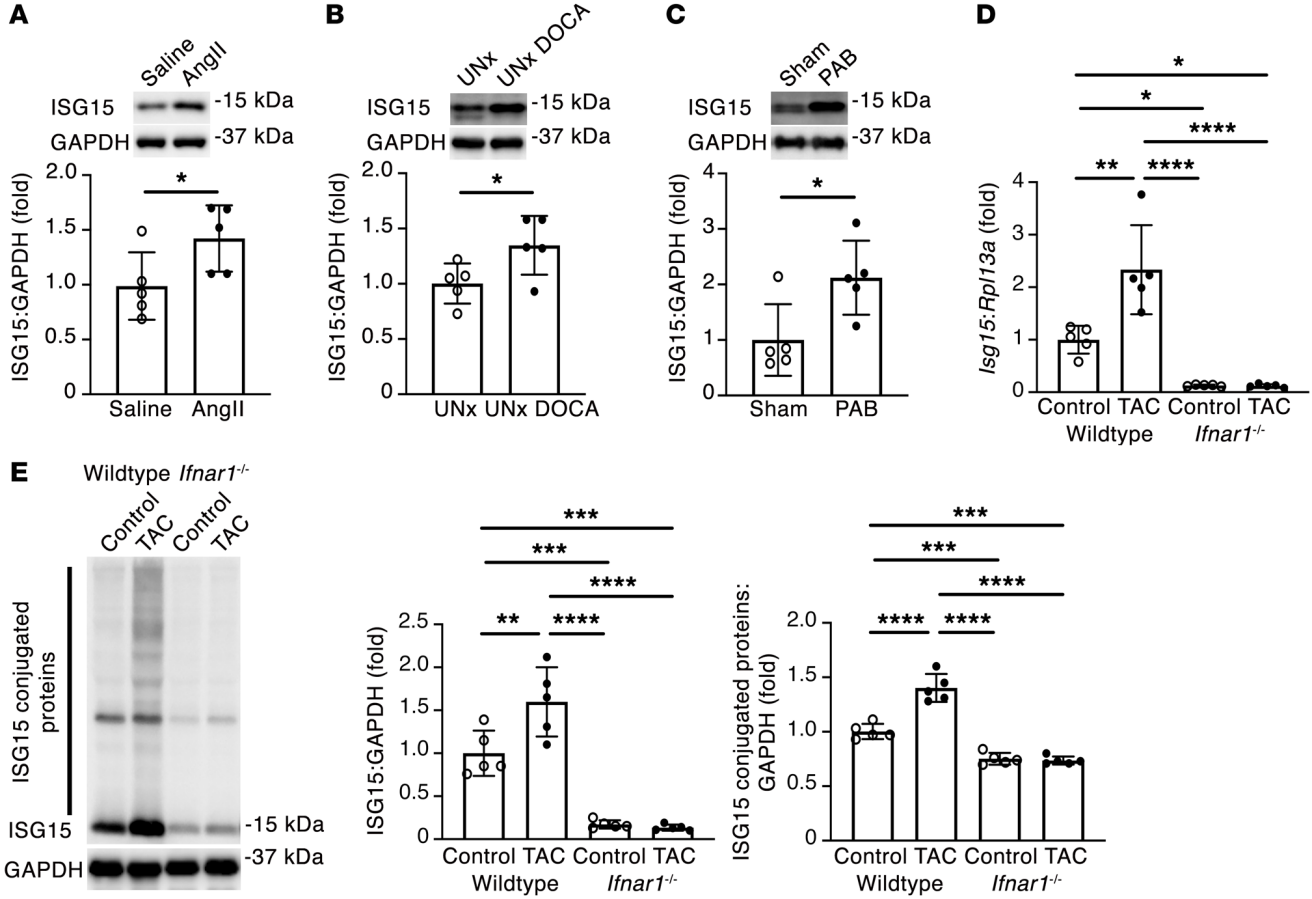
Pressure overload induces IFNAR-dependent ISG15 upregulation. (**A**) Immunoblotting for ISG15 in LV tissue of mice infused with Ang II (2 mg/kg/d) or saline for 14 days (*n* = 5 per group). (**B**) Immunoblotting for ISG15 in LV tissue of uninephrectomized rats (UNx) or UNx DOCA-salt rats followed for 4 weeks (UNx DOCA) (*n* = 5 per group). (**C**) Immunoblotting for ISG15 in right ventricular tissue of rats 6 weeks after sham or pulmonary artery banding (PAB) (*n* = 5 per group). (**D** and **E**) qRT-PCR (**D**) and immunoblotting (**E**) for ISG15 in WT and *Ifnar1^–/–^* mouse hearts 1 week after TAC (*n* = 5 per group). Values are mean ± SD. *P < 0.05, **P < 0.01, ***P < 0.001, ****P < 0.0001 by unpaired 2-tailed Mann-Whitney test (**A**), unpaired 2-tailed Student’s *t* test (**B** and **C**), or 1-way ANOVA followed by Tukey’s post hoc test (**D** and **E**).

Next, we validated the specificity of an anti-ISG15 antibody using protein isolated from the hearts of WT and *Isg15^–/–^* mice (24) (Figure 3A), also confirming that *Isg15^–/–^* mice are null mutants (henceforward, ISG15 deficient), and we confirmed by qRT-PCR (Figure 3B) and immunoblotting (Figure 3C) that ISG15 mRNA and protein levels were increased in mouse hearts after TAC. We similarly found that this upregulation in ISG15 was accompanied by an increase in ISG15 protein conjugates in TAC hearts (Figure 3D). To determine whether *Isg15* is expressed and upregulated by cardiomyocytes in vivo, we combined RNAscope in situ hybridization for *Isg15* with immunofluorescence staining for troponin I, observing *Isg15* RNAscope puncta in cardiomyocytes and in noncardiomyocyte cells, with a significant increase in cardiomyocyte *Isg15* transcript abundance following TAC (Figure 3E). By immunostaining, ISG15 was most readily observed in bands at, or near, intercalated discs in mouse hearts following TAC (Figure 3F). To determine whether cardiac upregulation of ISG15 occurs in other settings of pressure overload, we studied LV tissue from mice infused with angiotensin II (Ang II) and uninephrectomized rats implanted with deoxycorticosterone acetate (DOCA) and treated with 1% NaCl (UNx DOCA-salt) (25), as well as right ventricular tissue from rats after pulmonary artery banding (26). In each case, ISG15 protein levels were increased in pressure-overloaded rodent hearts (Figure 4, A–C).

Whereas type I IFNs induce ISG expression, they are not the only stimuli; cytosolic DNA- or RNA-sensing pattern recognition receptors (PRRs) are also implicated in ISG15 induction (12). By immunoblotting we observed a time-dependent upregulation of both cyclic GMP-AMP synthase (cGAS)/stimulator of IFN genes (STING) and retinoic acid–inducible gene I (RIG-I)/mitochondrial antiviral signaling protein (MAVS) after TAC (Supplemental Figure 8). Accordingly, to determine the extent to which cardiac ISG15 expression is dependent on type I IFN signaling, we performed TAC in *Ifnar1^–/–^* mice, lacking type I IFN receptor function (27). ISG15 levels were markedly lower in *Ifnar1^–/–^* mice than in WT mice (Figure 4, D and E), and ISG15 induction and protein ISGylation after TAC were abrogated in *Ifnar1^–/–^* mice (Figure 4, D and E). Therefore, cardiac ISG15 induction in pressure overload is primarily mediated by type I IFN signaling.

### ISG15 is inducible in mouse and human cardiomyocytes and is upregulated in human heart failure.

**Figure 5:**
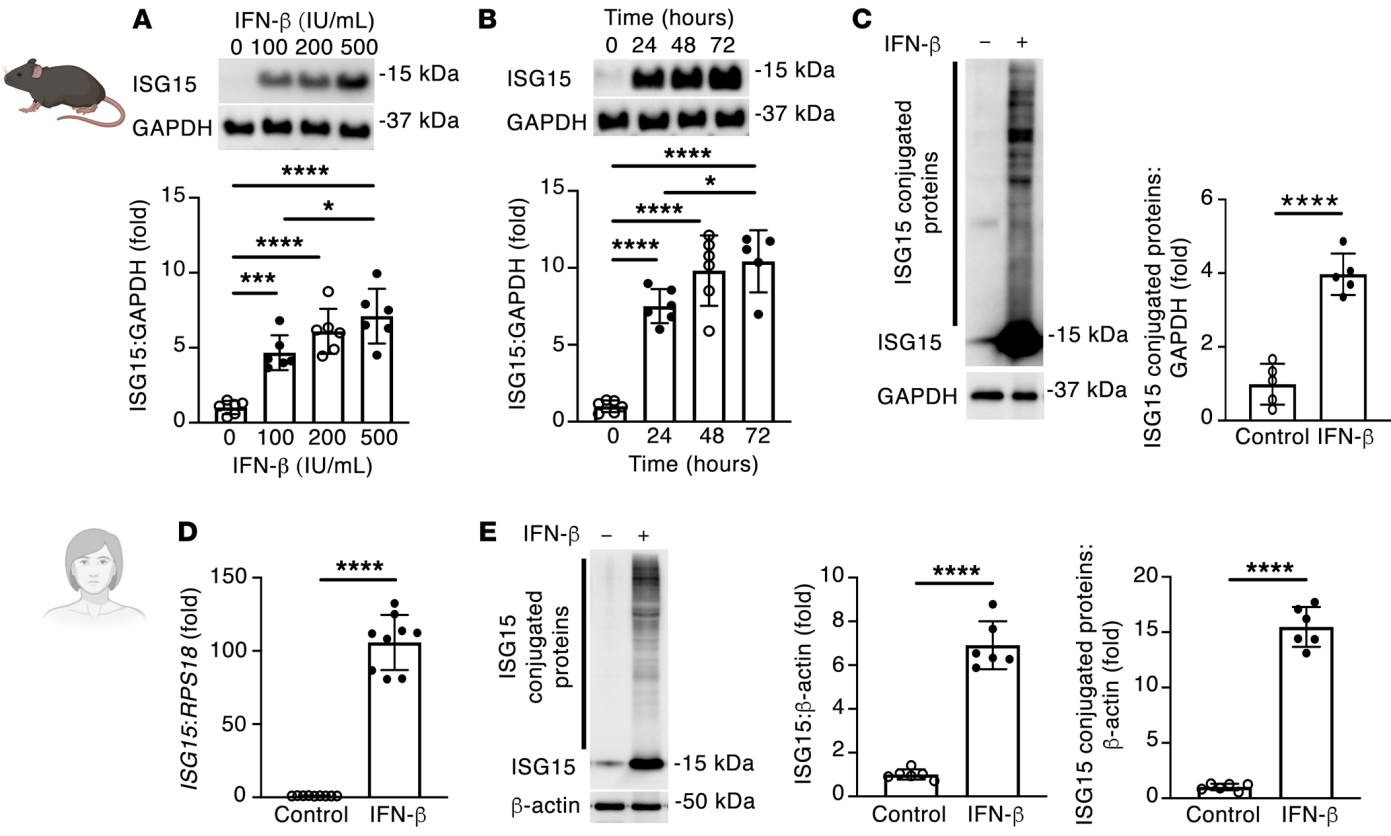
ISG15 is inducible in mouse and human cardiomyocytes. (**A**) ISG15 induction by recombinant IFN-β for 24 hours in primary mouse cardiomyocytes (*n* = 6 per condition). (**B**) ISG15 induction by 500 IU/mL recombinant IFN-β in primary mouse cardiomyocytes (*n* = 6 per condition, except 72 hours [*n* = 5]). (**C**) Immunoblotting for ISG15-conjugated proteins following stimulation with 500 IU/mL recombinant IFN-β for 48 hours (*n* = 5 per condition). (**D**) qRT-PCR for ISG15 in human cardiomyocytes incubated with 500 IU/mL IFN-β for 24 hours (*n* = 9 per condition). (**E**) Immunoblotting for ISG15 in human cardiomyocytes incubated with 500 IU/mL IFN-β for 48 hours (*n* = 6 per condition). Values are mean ± SD. *P < 0.05, ***P < 0.001, ****P < 0.0001 by 1-way ANOVA followed by Tukey’s post hoc test (**A** and **B**), or unpaired 2-tailed Student’s *t* test (**C**–**E**).

**Figure 6:**
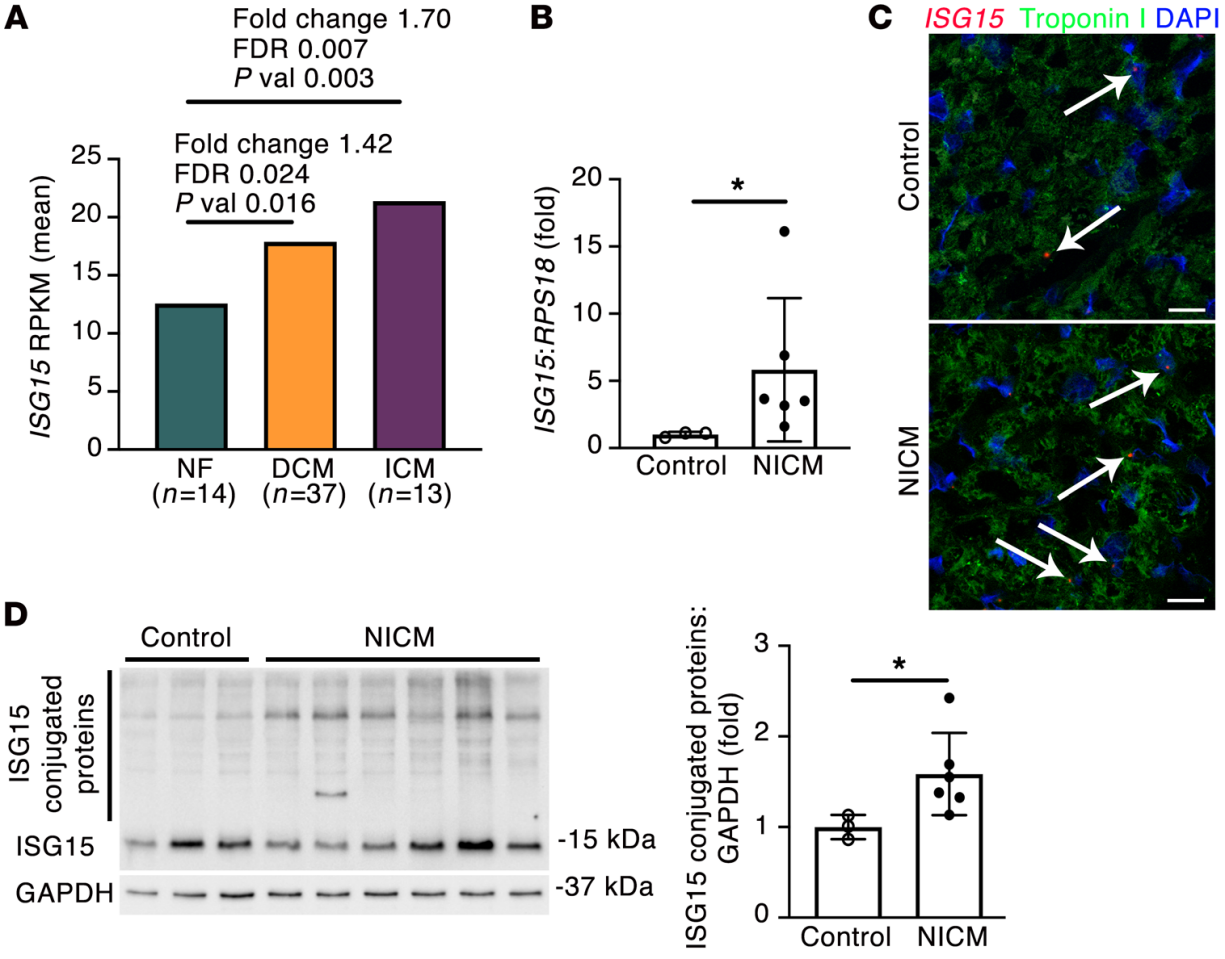
ISG15 is upregulated in human NICM. (**A**) *ISG15* in human LV samples from patients with nonfailing hearts (NF), dilated cardiomyopathy (DCM), or ischemic cardiomyopathy (ICM) (28). Differential expression determined by linear model ANOVA; *P* value (pval) adjusted for Benjamini-Hochberg FDR ≤ 0.05 (28). RPKM, reads per kilobase of transcript, per Mmllion mapped reads. (**B**) qRT-PCR in control human heart tissue (*n* = 3) and tissue from patients with end-stage heart failure due to NICM (*n* = 6). (**C**) RNAscope in situ hybridization for *ISG15* and immunofluorescence for troponin I in human LV tissue. The arrows mark *ISG15* RNAscope puncta in troponin I^+^ cardiomyocytes. Scale bars: 20 μm. (**D**) Immunoblotting for ISG15 and quantification of ISG15 protein conjugates in human control (*n* = 3) and NICM (*n* = 6) heart tissue. Values are mean ± SD. *P < 0.05 by unpaired 2-tailed Mann-Whitney test.

To confirm cardiomyocyte ISG15 induction by type I IFN signaling, we exposed primary adult mouse cardiomyocytes to recombinant IFN-β, observing a marked increase in ISG15 protein levels (Figure 5, A and B), accompanied by an increase in ISG15 protein conjugates (Figure 5C). Demonstrating that cardiomyocyte ISG15 induction is not solely regulated by IFN-β, however, cardiomyocyte ISG15 was also increased when cells were incubated with IFN-α or with poly(I:C) (Supplemental Figure 9). Next, we queried whether ISG15 induction and protein ISGylation also occur in human cardiomyocytes and whether *ISG15* upregulation also occurs in human nonischemic cardiomyopathy (NICM). Similar to what was observed in adult mouse cardiomyocytes, incubation of *GATA4*- and *ACTC1*-expressing human cardiac myocytes (mean ± SD Ct, *GATA4* 24.0 ± 0.2, *ACTC1* 27.2 ± 0.1, *RPL13A* 16.8 ± 0.5, *n* = 6; Supplemental Figure 10) with recombinant IFN-β caused a marked upregulation of ISG15 and a large increase in ISG15 protein conjugation (Figure 5, D and E). To determine whether ISG15 upregulation also occurs in human heart failure, we first explored previously published bioinformatic data. By RNA sequencing of 64 human LV samples comprising 14 nonfailing hearts, 37 LV samples from patients with dilated cardiomyopathy (DCM), and 13 LV samples from patients with ischemic cardiomyopathy (ICM), *ISG15* was found to be upregulated 1.4- to 1.7-fold in either DCM or ICM (Figure 6A) (28). To validate these initial in silico observations, we obtained tissue from patients with end-stage NICM at the time of cardiac transplantation or LV assist device implantation, and we compared this with tissue from the hearts of deceased organ donors (controls) (NICM [*n* = 7] mean age 53.3 ± 3 years, 7 male; control [*n* = 3] mean age 55.7 ± 8 years, 2 male, 1 female), observing an increase in *ISG15* mRNA (Figure 6B) and an increase in cardiomyocyte *ISG15* RNAscope puncta in NICM tissue (Figure 6C). By immunoblotting, this was associated with an increase in the abundance of ISG15 protein conjugation in NICM tissue (Figure 6D).

### The cardiomyocyte myofibrillar protein filamin-C is an ISGylation target.

**Figure 7:**
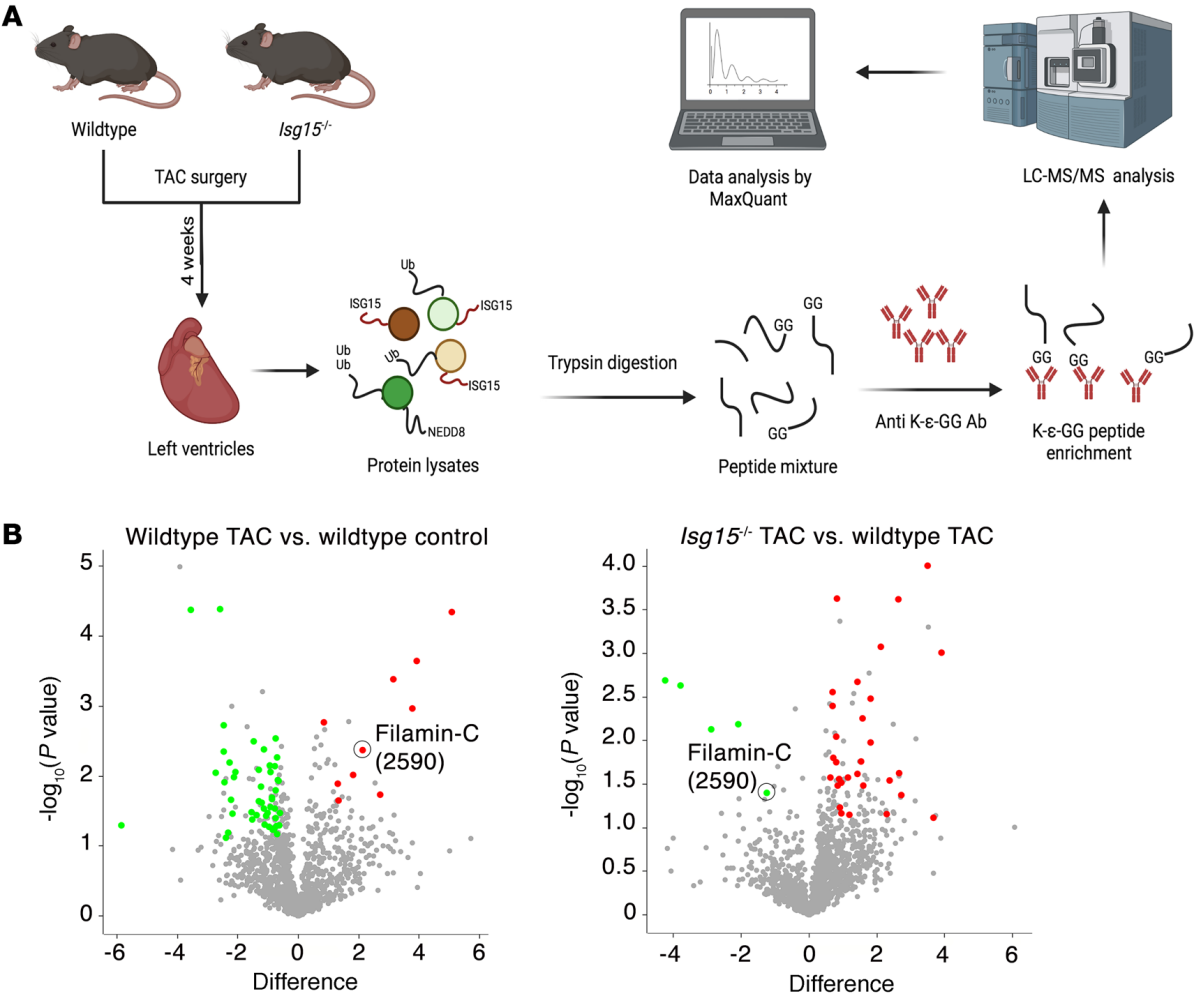
Identification of ISGylation targets in mouse hearts after TAC. (**A**) Design for diGLY proteomics experiments. (**B**) Volcano plots for the comparison of diGLY-enriched sites in WT TAC versus WT control and *Isg15^–/–^* TAC versus WT TAC (*n* = 3 per group). “Difference” indicates difference in the means of log_2_-transformed values between groups.

**Figure 8:**
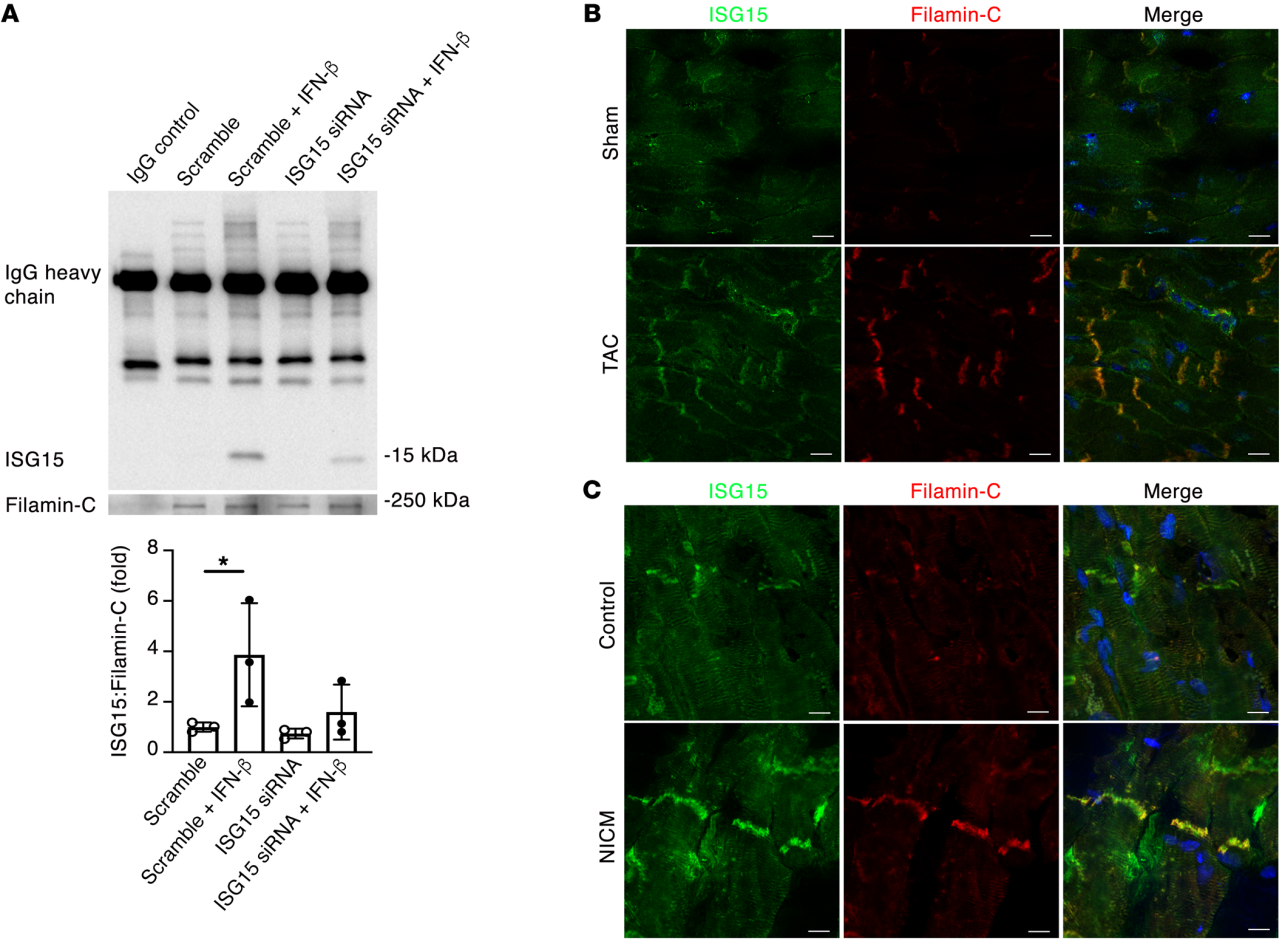
ISG15 associates with filamin-C in mouse and human hearts. (**A**) Immunoprecipitation for filamin-C and immunoblotting for ISG15 in human cardiomyocytes following knockdown of ISG15 with siRNA and incubation with 500 IU/mL IFN-β for 48 hours (*n* = 3 per condition). (**B** and **C**) Dual immunofluorescence staining for ISG15 and filamin-C in the hearts of sham-operated mice and mice 1 week after TAC (**B**) and human control tissue and LV tissue from a human with NICM (**C**). Scale bars: 10 μm. Values are mean ± SD. *P < 0.05 by 1-way ANOVA followed by Dunnett’s post hoc test.

We next set out to identify proteins that may be ISGylated in remodeling mouse hearts. To do this, we studied mouse hearts 4 weeks after TAC, a time point coinciding with an approximately 50% increase in LV mass (Supplemental Figure 11A), an approximately 2-fold increase in *Isg15* mRNA levels and ISG15-conjugated proteins (Figure 3, D and E), and the beginnings of echocardiographic evidence of impaired ventricular performance (Supplemental Figure 11, B–E). For our initial discovery experiments, we took advantage of the Ubl properties of ISG15, whereby trypsin cleavage of ISG15-conjugated proteins leaves a diglycine (GG) tag (diGLY) attached to the modified lysine of the ISGylated protein, analogous to that observed by conjugation of proteins to ubiquitin or the Ubl NEDD8 (neural precursor cell–expressed developmentally downregulated gene 8) (20, 29). Following diGLY peptide enrichment (Figure 7A), we identified 1,426 diglycine-modified lysine sites in 562 diGLY-tagged proteins. To identify proteins that are potentially ISGylated in the case of pressure overload, we focused on 10 protein candidates in which diGLY-tagged lysine residues were significantly upregulated in WT TAC mice (Supplemental Tables 7 and 8). We noted that several of these candidates are myofibrillar proteins (Xin actin-binding repeat–containing protein 2, titin, synaptopodin 2–like protein, filamin-C, and myosin-7) and that, of these 10 candidates, position 2590 of filamin-C was the only site that was also significantly downregulated in *Isg15^–/–^* TAC hearts in comparison with WT TAC hearts (Figure 7B). We also noted that filamin-C localized to regions that are exposed to increased mechanical stress, such as intercalated discs (30, 31). To verify that ISG15 associates with filamin-C in cardiomyocytes, we performed coimmunoprecipitation experiments in which IFN-β caused ISG15 to coimmunoprecipitate with filamin-C, with a reduction in this association following *ISG15* knockdown (Figure 8A). Lastly, using dual fluorescence immunostaining, we observed that ISG15 colocalized with filamin-C at, or close to, cardiomyocyte intercalated discs in the hearts of mice after TAC (Figure 8B) and humans with NICM (Figure 8C), with an expected absence of ISG15 colocalizing with filamin-C in *Isg15^–/–^* mice (Supplemental Figure 12).

### ISG15 deficiency preserves LV function in pressure-overloaded mouse hearts and improves recovery of mouse hearts ex vivo.

**Figure 9:**
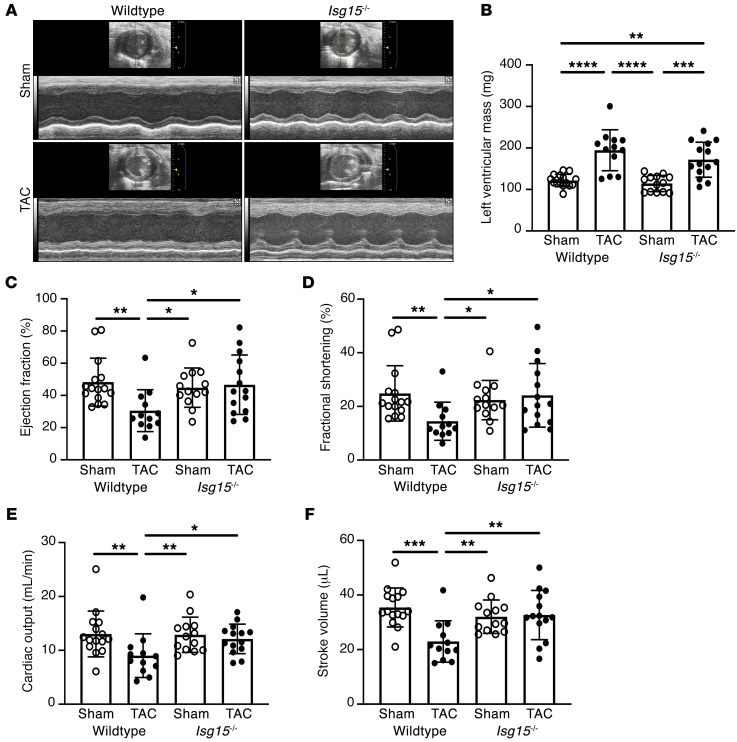
ISG15 deficiency attenuates LV systolic dysfunction in mice after TAC. (**A** and **B**) Representative M-mode echocardiographs (**A**) and LV mass (**B**) in WT and *Isg15^–/–^* mice 8 weeks after sham or TAC surgery. (**C**–**F**) Ejection fraction (**C**), fractional shortening (**D**), cardiac output (**E**), and stroke volume (**F**) in WT and *Isg15^–/–^* mice 8 weeks after sham or TAC. WT sham, *n* = 15; WT TAC, *n* = 12; *Isg15^–/–^* sham, *n* = 13; *Isg15^–/–^* TAC, *n* = 14. Values are mean ± SD. *P < 0.05, **P < 0.01, ***P < 0.001, ****P < 0.0001 by 1-way ANOVA followed by Tukey’s post hoc test (skew-distributed data in **B**–**F** were log-transformed before statistical comparison).

To determine whether ISG15 expression or upregulation contributes to cardiac dysfunction, we subjected WT and *Isg15^–/–^* mice to pressure overload induced by TAC (Supplemental Figure 13 and Supplemental Tables 9–11). After 8 weeks, heart weight/tibia length ratio was increased in both WT and *Isg15^–/–^* TAC mice in comparison with sham-operated animals, although it was marginally lower in *Isg15^–/–^* TAC mice than in WT TAC mice (Supplemental Table 9). LV mass, however, was increased in both *Isg15^–/–^* TAC mice and WT TAC mice, whereas mean LV mass was numerically but nonsignificantly lower with ISG15 deficiency (Figure 9, A and B). In contrast, ejection fraction, cardiac output, fractional shortening, and stroke volume were all reduced 8 weeks after TAC in WT mice, yet each of these parameters was comparatively preserved in ISG15-deficient mice 8 weeks after TAC (Figure 9, C–F). In contrast, myocyte cross-sectional area (Supplemental Figure 14A) and interstitial collagen deposition (Supplemental Figure 14B) were increased equivalently in WT and *Isg15^–/–^* mice after TAC, whereas mitochondrial density was equivalently decreased (Supplemental Figure 14C).

**Figure 10:**
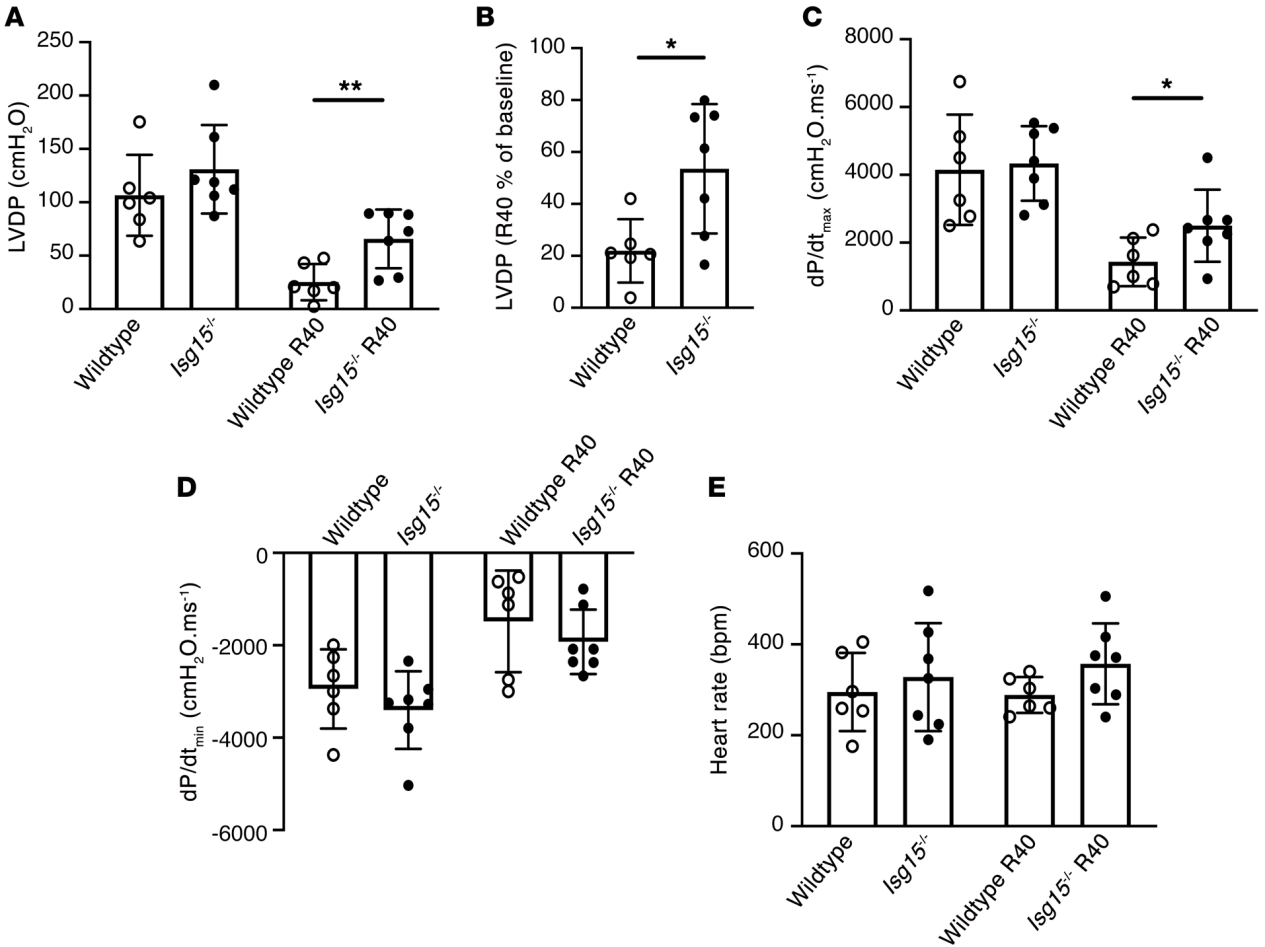
ISG15 deficiency improves contractile recovery of isolated mouse hearts. (**A**) Left ventricular developed pressure (LVDP) at baseline and 40 minutes after ischemia/reperfusion (R40) in isolated perfused hearts from WT (*n* = 6) and *Isg15^–/–^* (*n* = 7) mice. (**B**) Percentage recovery of LVDP 40 minutes after reperfusion. (**C**) *dP/dt*_max_. (**D**) *dP/dt*_min_. (**E**) Heart rate. Values are mean ± SD. *P < 0.05, **P < 0.01 by unpaired 2-tailed Student’s *t* test (**A** and **B**) or unpaired 2-tailed Mann-Whitney test (**C**).

Mindful that ISG15 absence from immune cells of *Isg15^–/–^* mice could affect their recruitment to the remodeling heart, we used dual immunofluorescence to study the time course of *Ccr2*^+^ cell accumulation in the hearts of ISG15-deficient mice after TAC. As had previously been observed in WT and *Ccr2^gfp/+^* mice (Figure 1, A and B), there was an early (within 1 week) accumulation of *Ccr2*^+^ cells in *Isg15^–/–^* mouse hearts after TAC, gradually declining after that (Supplemental Figure 15). To further examine the effects of ISG15 in the absence of immune cell infiltration, and cognizant from our studies in *Ifnar1^–/–^* mice of constitutive ISG15 expression and protein ISGylation (Figure 4, D and E), we isolated the hearts of WT and *Isg15^–/–^* mice and perfused them ex vivo. Whereas basal cardiac function did not differ between WT and *Isg15^–/–^* mouse hearts, following 20 minutes of ischemia and 40 minutes of reperfusion, left ventricular developed pressure (LVDP) (Figure 10A), recovery of LVDP (Figure 10B), and *dP/dt*_max_ (Figure 10C) were improved in *Isg15^–/–^* mouse hearts in comparison with WT mouse hearts, with no difference observed in *dP/dt*_min_ or heart rate (Figure 10, D and E).

### ISG15 regulates cardiac amino acid metabolism and cardiomyocyte protein quality control.

**Figure 11:**
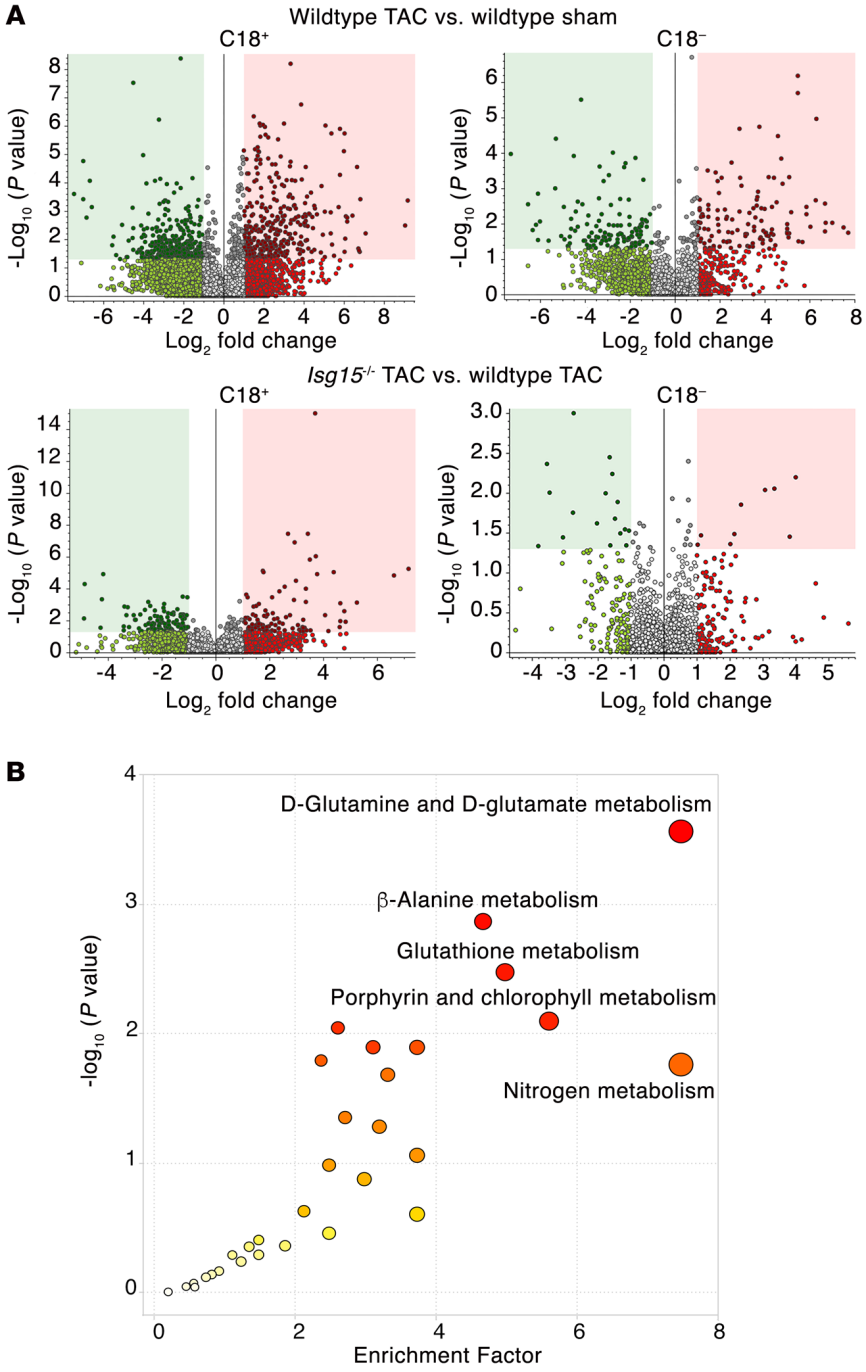
ISG15 deficiency alters amino acid metabolism in pressure-overloaded mouse hearts. (**A**) Volcano plots of untargeted metabolomic comparison of WT and *Isg15^–/–^* mouse hearts 8 weeks after sham or TAC surgery. Top: WT TAC hearts (*n* = 4) versus WT sham (*n* = 3). Bottom: *Isg15^–/–^* TAC hearts (*n* = 4) versus WT TAC (*n* = 4). (**B**) KEGG pathway analysis of metabolic pathways enriched in *Isg15^–/–^* mouse hearts versus WT mouse hearts 8 weeks after TAC. Enrichment factor = ratio of significant pathway hits versus expected pathway hits (*n* = 4 per group).

**Figure 12:**
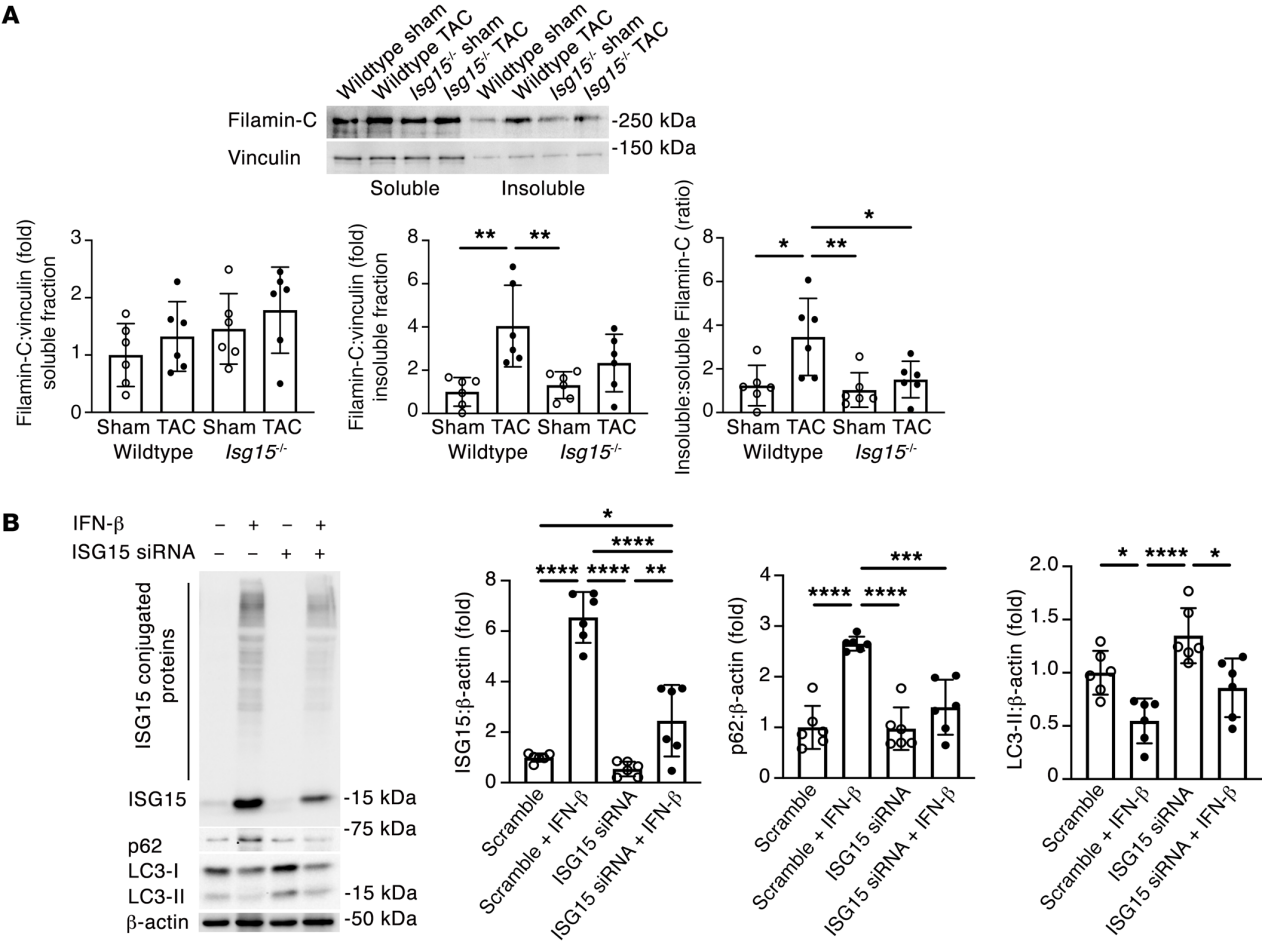
ISG15 induction impairs cardiomyocyte protein turnover. (**A**) Immunoblotting for filamin-C in the soluble and insoluble fractions of WT and *Isg15^–/–^* mouse hearts 8 weeks after sham or TAC surgery (*n* = 6 per group). (**B**) Immunoblotting for ISG15, ISG15-conjugated proteins, p62, and LC3 in human cardiomyocytes transfected with siRNA directed against ISG15 for 6 hours before incubation with 500 IU/mL IFN-β for 48 hours. Values are mean ± SD. *P < 0.05, **P < 0.01, ***P < 0.001, ****P < 0.0001 by 1-way ANOVA followed by Tukey’s post hoc test.

Finally, having observed the preservation of cardiac function in *Isg15^–/–^* mice after TAC and in mouse hearts following ex vivo ischemia/reperfusion, we explored possible mechanisms underlying the cardioprotective effect of ISG15 absence. Literature review indicated to us that ISG15 and protein ISGylation have predominant influence over cellular metabolic processes and protein quality control (20, 32–35), the dysregulation of which has also been previously linked to cardiac dysfunction (36, 37). Accordingly, we set out to determine whether cardiac metabolite levels in pressure-overloaded hearts are altered in the absence of ISG15. We therefore performed untargeted metabolomics of the hearts of sham and TAC WT and *Isg15^–/–^* mice (Figure 11A), observing group separation on principal component analysis plots (Supplemental Figure 16). KEGG pathway analysis of the comparison of *Isg15^–/–^* and WT mouse hearts after TAC revealed differential enrichment of 12 pathways in positive-ion mode and no pathways in negative-ion mode (Figure 11B and Supplemental Table 12). Enriched pathways included d-glutamine and d-glutamate metabolism, β-alanine metabolism, glutathione metabolism, and nitrogen metabolism (Figure 11B and Supplemental Table 12), suggestive of differential amino acid/protein metabolism in WT TAC hearts and *Isg15^–/–^* TAC hearts. Because of the relationship between changes in amino acid levels and protein turnover (38, 39), the importance of protein turnover to sarcomeric function (37), and a possible role for ISGylation in regulating protein turnover (35, 40), we hypothesized that ISG15 and its binding to cardiomyocyte proteins could influence their aggregation and/or clearance. Consistent with such a role, when we immunoblotted mouse hearts for filamin-C, we observed an increase in filamin-C in the insoluble fraction of WT TAC hearts that was attenuated with ISG15 deficiency, indicative of filamin-C aggregation in the presence of ISG15 (Figure 12A). We thus queried whether ISG15 induction and ISG15 knockdown influence cardiomyocyte protein quality control processes themselves. As expected, siRNA directed against *ISG15* attenuated ISG15 upregulation in human cardiomyocytes exposed to IFN-β (Figure 12B). Interestingly, IFN-β caused the adaptor protein p62 to accumulate in human cardiomyocytes, accompanied by a reduction in the autophagy marker LC3-II, consistent with impaired autophagy induction (Figure 12B). In contrast, *Isg15* siRNA increased LC3-II and, in the presence of IFN-β, prevented p62 accumulation indicative of cardiomyocyte autophagy induction with ISG15 knockdown (Figure 12B).

## Discussion

In the present study, we set out to uncover ways in which immune cells recruited to the heart during ventricular remodeling affect cardiomyocyte function. Over the course of this work, we discovered that the Ubl ISG15 is constitutively expressed by cardiomyocytes, and it is induced in cardiomyocytes during pressure overload as part of a type I IFN response. ISG15 posttranslationally modifies actively translated cardiomyocyte proteins, regulates cardiomyocyte protein turnover, and limits cardiac performance under stressed conditions. Thus, ISG15 induction and protein ISGylation are intracellular mechanisms that provide a mechanistic link between sterile inflammation, adverse ventricular remodeling, and eventual heart failure development.

Although we arrived at our discovery of the actions of ISG15 in ventricular remodeling by first examining the effects of CCR2^+^ cardiac macrophages in mouse hearts with TAC-induced pressure overload, CCR2^+^ cardiac macrophages are not the sole potential inducers of ISG15. For instance, dendritic cells (41), mast cells (42), T cells (43), B cells (44), and neutrophils (45) have each also been implicated in the pathogenesis of hypertrophic heart failure, and in our own experiments CD4^+^ T cells similarly induced cardiomyocyte ISG15 upregulation. Likewise, the secretory products of macrophages include many biologically active substances that could trigger a damage response in neighboring cells (46). However, we observed lower levels of ISG15 in the hearts of *Ifnar1^–/–^* mice with TAC, indicating that the primary inducer of ISG15 in pressure-overloaded cardiomyocytes is canonical type I IFN signaling. Interestingly, ISG15 levels were also markedly diminished in *Ifnar1^–/–^* mouse hearts even under basal conditions. This finding is aligned with the notion that, although they are present at very low concentrations, constitutively expressed type I IFNs have important physiological roles (47). In the heart, these physiological roles appear to include the control of constitutive ISG15 expression.

ISG15 exists in 3 forms: an intracellular free form, an intracellular form that is conjugated to target proteins, and an extracellular secreted form (48). The secreted form of ISG15 binds to its extracellular receptor, leukocyte function–associated antigen-1 (LFA-1) (49), enhancing the secretion of IFN-γ, which is important for the innate and adaptive immune response (50, 51). The main function of intracellular ISG15 appears, however, to be the posttranslational modification of viral and cellular proteins, analogous to ubiquitylation. Proteolytic processing of the 17 kDa precursor form of ISG15 to its mature 15 kDa form exposes a carboxy-terminal LRLRGG motif that allows the covalent conjugation of ISG15 to lysine residues of target proteins (52). Like the ubiquitin conjugation system, ISGylation entails an energy-consuming 3-step process involving E1-activating enzymes, E2-conjugating enzymes, and E3-ligating enzymes (48, 53), whereas the process of ISGylation is reversed by an ISG15-specific protease, ubiquitin-specific protease 18 (USP18) (54). We took advantage of the ubiquitin-like properties of ISG15 and performed quantitative diGLY proteomics to identify possible ISGylated proteins in mouse hearts after TAC (20, 29). It is important to note that this approach cannot distinguish ISGylated peptides from ubiquitylated or even NEDDylated peptides. Indeed, one previous study employing diGLY proteomics concluded that more than 94% of identified sites represent ubiquitin conjugation rather than ISG15 or NEDD8 conjugation (55). Thus, most of the 1,426 sites we identified during this initial discovery experiment will be sites of ubiquitylation. Within these, however, will reside some sites that are ISGylated. We reasoned that possible candidates involved in the pathogenesis of pressure overload–induced ventricular remodeling would be expected to be differentially upregulated in WT TAC hearts in comparison with controls. Interestingly, several of the candidates that satisfied this minimum criterion were myofibrillar proteins that are newly synthesized during cardiomyocyte hypertrophy (31). We focused on filamin-C because this protein contained the sole upregulated diGLY site that was significantly downregulated in *Isg15^–/–^* TAC hearts, rendering it most likely to be ISGylated; and the association of ISG15 with filamin-C was confirmed by coimmunoprecipitation. Filamin-C is an actin-binding protein that is localized to the Z disc periphery, costameres, and intercalated discs in cardiomyocytes and is important for sarcomere stability, mechanical stabilization, mechanosensation, and intracellular signaling (56, 57). Mutations in filamin-C have been linked to hypertrophic cardiomyopathy (58, 59), and its inducible knockout from cardiomyocytes causes a rapid decline in LV function (60). Like ISG15, filamin-C also localizes at, or close to, intercalated discs during TAC-induced ventricular remodeling (31). In the present study, we observed that ISG15 and filamin-C colocalized to intercalated discs in pressure-overloaded mouse hearts and in the hearts of humans with NICM.

Whereas there are several different mechanisms by which protein ISGylation may affect cellular function, our metabolomics experiments indicate that the primary effects of ISG15 in pressure overload may be mediated by alterations in protein turnover. This could occur at the level of the individual ISGylated protein, at the level of bulk protein quality control processes, or both. For instance, we observed that filamin-C aggregated in TAC hearts in an ISG15-dependent manner. Posttranslational modifications can promote protein misfolding and impair heart function (61), and filamin-C variants that promote misfolding can saturate the ubiquitin-proteasome system (UPS) and autophagy-lysosome pathways (62). ISG15 may also compete with ubiquitin for ubiquitin-binding sites, thus impairing clearance of aggregated filamin-C by the UPS (12). We also observed that *Isg15* knockdown promoted cardiomyocyte autophagy. ISGylation has previously been linked to both increases and decreases in flux through the UPS (63, 64) and autophagy pathways (20, 40). In the heart, sarcomeric protein turnover is dependent on chaperone-assisted selective autophagy (CASA) (37). Accordingly, it is possible that ISG15 and ISG15-conjugating enzymes compete for substrate binding with E3 ubiquitin ligases necessary for CASA (37), thus impairing the bulk degradation of misfolded sarcomeric proteins and restricting cardiac performance. Distinct from these mechanisms, ISG15 may also posttranslationally modify the autophagy machinery itself (40). In short, there are multiple complementary pathway steps whereby ISG15 and protein ISGylation may affect protein turnover, and we posit that, in pressure overload, preferential ISGylation of newly synthesized myofibrillar proteins facilitates protein aggregation, impairing cardiac contractility.

Our study has limitations. Firstly, it has been suggested that ISG15 has different effects in mice and humans (17). This difference may be due to divergent actions of human and murine ISG15 on the stability of USP18, the major isopeptidase that cleaves ISG15 from conjugates (65). In addition to removing ISG15 conjugates, USP18 also limits IFN signaling by preventing dimerization of IFNAR subunits (17). In humans, binding of ISG15 to USP18 prevents USP18 degradation, amplifying the inhibition of IFN signaling. However, in mice USP18 is not stabilized by ISG15 (17). In our studies, IFN-β caused a marked upregulation of ISG15 and of protein ISGylation in cultured human cardiomyocytes. In human NICM tissue, upregulated ISG15 was largely present in its intracellular protein-conjugated form. Interestingly, *Usp18* overexpression, which would be expected to blunt the accumulation of ISG15-conjugated proteins, has also been reported to attenuate pathological remodeling in mice (66). Thus, the collective body of evidence in mice and humans in the present study and parallel evidence from *Usp18*-overexpressing mice supports a role for protein ISGylation in the pathogenesis of adverse ventricular remodeling. Secondly, at first glance the findings herein reported may appear to be at variance with those observed in the setting of viral myocarditis where ISG15 induction was reported to preserve cardiomyocyte health (15). However, this discordance is to be expected. Being localized to the polyribosome (53), ISG15 protein ligases primarily target actively translated proteins (17). In the absence of viral infection, but in the presence of pressure overload and compensatory cardiomyocyte enlargement, ISG15 conjugates to actively translated cellular proteins. Thirdly, although our proteomic experiments and in vitro experiments point to a primary role for ISG15 in regulating cardiomyocyte function in pressure overload, it is possible (and indeed likely) that ISG15 has important effects in other cardiac cell types as well. For instance, a recent report demonstrated that ISG15 mediates endothelial dysfunction and aneurysm formation in mice infused with Ang II (67). ISG15 has also been reported to influence the actions of macrophages (34). In the present study, we observed that BMDMs from *Isg15^–/–^* mice responded similarly to LPS compared with their WT counterparts, that ISG15 deficiency did not affect *Ccr2*^+^ cell recruitment to pressure-overloaded hearts, and that ISG15 deficiency enhanced cardiac recovery ex vivo. Lastly, whereas we arrived at the study of ISG15 because of its marked induction in cardiomyocytes, constitutive ISG15 may itself also exert subtle but important effects on cardiomyocyte function. Cardiac ISG15 levels are lower in *Ifnar1^–/–^* mice, and although resting cardiac function does not differ between WT and *Isg15^–/–^* mice, recovery of contractile function after ischemia/reperfusion was enhanced in the absence of ISG15. The relative short time frame over which this ex vivo effect occurred is suggestive of a priming influence of ISG15 absence, and, in vitro, *ISG15* knockdown increased basal cardiomyocyte autophagy. These limitations notwithstanding, in recent years several posttranslational modifications have been linked to pathological cardiac hypertrophy, including phosphorylation, ubiquitylation, acetylation, methylation, SUMOylation, and O-GlcNAcylation (68). The present study now lays the foundation to add ISGylation to this list as an inflammation-driven posttranslational modification important in the pathogenesis of ventricular remodeling and in the regulation of cardiac function under stressed conditions. Future studies may build on this foundation to uncover the relative contributions of free, protein-bound, and secreted ISG15 in cardiomyocytes and noncardiomyocytes, in ischemic and nonischemic remodeling, and under basal and induced conditions.

In summary, pressure overload–induced ventricular remodeling is accompanied by an IFN response that is characterized by induction of the Ubl ISG15 and the conjugation of ISG15 to actively translated cardiomyocyte proteins, facilitating LV functional decline.

## Methods

### Animal studies.

*Ccr2^–/–^* mice (B6.129S4-*Ccr2^tm1Ifc^*/J, stock 004999), *Ccr2^gfp/gfp^* KI/KO mice [B6(C)-*Ccr2^tm1.1Cln^*/J, stock 027619], *Isg15^–/–^* mice (B6.129P2-*Isg15^tm1Kpk^*/J, stock 010486), *Ifnar1^–/–^* mice [B6(Cg)-*Ifnar1^tm1.2Ees^*/J, stock 028288], and C57BL/6J mice (stock 000664) were obtained from The Jackson Laboratory. Males aged approximately 8–12 weeks were studied. TAC surgeries were performed as previously described (69, 70). Systolic blood pressure was recorded using a CODA noninvasive blood pressure system (Kent Scientific) (71). Transthoracic echocardiography was performed under 1% isoflurane using a high-frequency ultrasound system (Vevo 2100, MS550D transducer, Visual Sonics Inc.). For invasive hemodynamic monitoring, a 1.4F pressure-volume catheter (Millar Mikro-Tip, AD Instruments) was inserted into the right carotid artery and advanced into the LV. Data were acquired and recorded using the MPVS Ultra data acquisition system (AD Instruments). Functional parameters were calculated using LabChart Pro (AD Instruments) (72). For Ang II infusion, mice were implanted with subcutaneous osmotic minipumps (1002, ALZET) and infused with Ang II (2 mg/kg/d in 0.9% saline) or 0.9% saline alone for 14 days (73). For DOCA-salt studies, Sprague-Dawley rats underwent unilateral nephrectomy and were followed for 4 weeks after being implanted with 200 mg DOCA in silicone rubber, with 1% saline as drinking water, as previously described (25). Pulmonary artery banding experiments were performed in Fischer F344 rats as previously described, with animals followed for 6 weeks (26). For Langendorff studies, mouse hearts were mounted on a Radnoti Langendorff Constant Pressure Apparatus (AD Instruments) and were perfused at constant pressure with continuously oxygenated Krebs-Henseleit buffer at 37°C as previously described (74). LVDP, *dP/dt*_max_, *dP/dt*_min_, and heart rate were measured and analyzed at baseline and 40 minutes after reperfusion (following 20 minutes of no-flow ischemia [R40]) using PowerLab 8/35 and LabChart Pro (AD Instruments).

### In situ hybridization and immunohistochemistry.

In situ hybridization was performed with RNAscope (Advanced Cell Diagnostics) according to the manufacturer’s instructions and using custom software as previously described (75), with probe sets specific for *Ccr2* (433271) and *ISG15* (mouse 559271; human 467741). For *Ccr2*^+^ cells or cardiomyocyte *ISG15* staining, after RNAscope in situ hybridization, tissue sections were immunostained with anti–troponin I antibody (1:200 dilution; ab47003, Abcam) and secondary antibody Alexa Fluor 647–donkey anti-rabbit (1:100 dilution; A31573, Thermo Fisher Scientific). DAPI was from Cell Signaling Technology and was used at 1:10,000 dilution. Slides were visualized with a Zeiss LSM 700 confocal microscope (Carl Zeiss Canada). *Ccr2*^+^ cells were quantified on each heart section using digitized images (×20 magnification, Zeiss AxioScan.Z1), with Zeiss Zen Blue software (×8 zoom). Cardiomyocyte *Isg15* RNAscope puncta were manually counted in a masked manner in 6 different, randomly selected fields using the annotation function in the HALO image analysis platform (Indica Labs). Immunohistochemistry for ISG15 was performed on mouse heart sections using a rabbit polyclonal antibody at 1:100 dilution (PA5-88262, Thermo Fisher Scientific).

### Flow cytometry.

*Ccr2^gfp/+^* mice (76) underwent TAC surgery as already described. Flow cytometry was performed using a method adapted from that reported by Dick et al. (77), with labeling of cells using an antibody cocktail shown in Supplemental Table 13. CCR2^+^ monocyte-derived macrophages were identified as CD45^+^Ly6C^hi^CD11b^+^CD64^+^MHC-II^+^GFP^+^ cells using a BD LSR Fortessa-X20 (BD Biosciences). Data were later analyzed using FlowJo software (FlowJo LLC).

### Adult mouse cardiomyocyte isolation.

Primary cultured cardiomyocytes were isolated from adult mouse hearts following the protocol described by Ackers-Johnson et al. (78). Cells were treated with recombinant mouse IFN-β or IFN-α (R&D Systems) or with poly(I:C) LMW (low molecular weight)/LyoVec (InvivoGen) at the concentrations and durations reported.

### Isolation of CCR2^+^ cardiac macrophages and exposure of cardiac myocytes to conditioned medium.

CCR2^+^ monocyte-derived macrophages (CD45^+^Ly6C^hi^CD11b^+^CD64^+^MHC-II^hi^GFP^+^ cells) were isolated from the hearts of TAC mice and sorted into M199 medium (Thermo Fisher Scientific) with 15% FBS using a BD FACSAria III Cell Sorter (BD Biosciences). Thirty thousand to forty thousand cells were cultured for 24 hours before collection of the medium, which was then supplemented with 10 μmol/L (–)-Blebbistatin (Cayman Chemical), Insulin-Transferrin-Selenium (ITS) (Thermo Fisher Scientific), and CD lipid (MilliporeSigma). Primary cultured mouse cardiomyocytes were incubated with CCR2^+^ cardiac macrophage–conditioned medium, or M199 medium alone, supplemented with (–)-Blebbistatin, ITS, and CD lipid for 24 hours before isolation of RNA with TRIzol reagent (Thermo Fisher Scientific). Cytokine content of CCR2^+^ cardiac macrophage–conditioned medium was determined with the Mouse Cytokine 44-Plex Discovery Assay (MECY2MAG-73K, Eve Technologies). IFN-α concentrations were assessed using high-sensitivity ELISA (PBL Assay Science, 42115-1).

### RNA sequencing.

RNA sequencing was performed using the 6G RNA Sequencing Service (150 bp paired-end, 40 million reads) from ArrayStar. Sequencing was performed on an Illumina Novaseq 600 (150 cycles for both ends). Solexa pipeline v1.8 was used for image analysis and base calling, and FastQC was used for examination of sequence quality. Hisat2 software was used to align trimmed reads (5′-, 3′-trimmed adaptor bases using cutadapt) to the GRCm38 reference genome (8). Transcript abundances were estimated using StringTie (79), and the FPKM and differential gene expression were determined using the R package Ballgown (80). Volcano plots, heatmaps, Gene Ontology, and Pathway Analysis were generated with the differentially expressed genes in R, Python, or shell environment (74). Data were deposited in the NCBI’s Gene Expression Omnibus database (accession number GSE196798).

### Quantitative reverse transcription PCR.

cDNA was reverse transcribed from 1 μg RNA using a High Capacity cDNA Reverse Transcription Kit (Thermo Fisher Scientific). Primer sequences were from Integrated DNA Technologies and are listed in Supplemental Table 14. SYBR Green–based qRT-PCR was performed on a QuantStudio 7 Flex Real-Time PCR System (Thermo Fisher Scientific). Data analysis was performed using Applied Biosystems Comparative CT method.

### Bone marrow–derived macrophages.

Bone marrow cell culture was based on the protocol described by Toda et al. (81). Briefly, bone marrow cells were collected by flushing of the femurs and tibiae of *Ccr2^gfp/+^* and *Isg15^–/–^* mice with DMEM. Bone marrow stem cells were allowed to differentiate in phenol red–free (high-glucose) DMEM containing 10% FBS, 10 ng/mL M-CSF (M9170, MilliporeSigma), and 1% penicillin-streptomycin for 7 days. The macrophage population was determined by flow cytometry for CD45, Ly6C, CD11b, CD64, MHC-II, F4/80 (53-4801-82, Thermo Fisher Scientific), and CCR2-GFP using an SP6800 Sony Spectral Analyzer (Sony Biotechnology). For determination of IFN-β secretion, BMDMs were serum-starved for 16 hours, with medium replaced with culture medium without M-CSF and containing poly(I:C) (LMW)/LyoVec (500 ng/mL), STING agonist-4 (5 μmol/L; HY-123943, MedChemExpress), or LPS (1 μg/mL; MilliporeSigma). After 24 hours, IFN-β concentrations in culture medium were determined by ELISA (42410, PBL Assay Science).

### CD4^+^ T cell isolation.

CD4^+^ T cells were isolated from mouse spleens using an EasySep Mouse CD4^+^ T cell Isolation Kit (STEMCELL Technologies). The CD4^+^ cell population was determined after staining of cells with anti-CD4 (RM4-5) rat mAb (PerCP-Cy5.5 conjugate) (49482, Cell Signaling Technology) using an SP6800 Sony Spectral Analyzer. Primary mouse cardiomyocytes were incubated in medium conditioned by CD4^+^ T cells for 24 hours.

### Immunoblotting.

Immunoblotting was performed with antibodies against the following proteins: ISG15 (1:1,000; mouse, 703132, clone 1H9L21; human and rat, PA5-88262; Thermo Fisher Scientific), GAPDH (1:1,000; 2188, Cell Signaling Technology), cGAS (1:1,000; 31659, Cell Signaling Technology), STING (1:1,000; 13647, Cell Signaling Technology), RIG-I (1:1,000; 3743, Cell Signaling Technology), MAVS (1:1,000; 4983, Cell Signaling Technology), β-actin (1:10,000; A1978, MilliporeSigma), filamin-C (1:1,000; NBP1-89300, Novus Biologicals), vinculin (1:1,000; 4650, Cell Signaling Technology), p62 (1:1,000; 109012, Abcam), and LC3 (1:1,000; 12741, Cell Signaling Technology). Immunoblotting for the soluble and insoluble fractions of filamin-C was performed in mouse hearts as previously described (82). Densitometry was performed using ImageJ version 1.39 (NIH).

### Human cardiac myocytes.

Human cardiac myocytes (PromoCell) were studied, at 60%–70% confluence, after a minimum of 21 days and after confirmation of *GATA4* and *ACTC1* expression by qRT-PCR. Cells were treated with 500 IU/mL recombinant IFN-β for 48 hours (R&D Systems). For knockdown experiments, cells were incubated with a mixture of siRNA directed against ISG15 or negative control siRNA (MilliporeSigma) at 50 nM concentration and Lipofectamine RNAiMAX Transfection Reagent (Thermo Fisher Scientific) for 6 hours before incubation with 500 IU/mL IFN-β for 48 hours. Coimmunoprecipitation was performed using Protein G magnetic beads (Cell Signaling Technology) with either an anti–filamin-C antibody (1:50; 86972, Cell Signaling Technology) or isotype IgG (1:50; ab172730, Abcam) before immunoblotting for ISG15 (1:1,000; PA5-88262, Thermo Fisher Scientific) or filamin-C (1:1,000; NBP1-89300, Novus Biologicals).

### Human studies.

LV tissue was obtained from deceased organ donors (control, *n* = 3) or patients with end-stage NICM (*n* = 7) at the time of LV assist device implantation or cardiac transplantation. Six NICM and 3 control samples were studied each for qRT-PCR and immunoblotting experiments.

### diGLY proteomics.

diGLY proteomics was performed by Creative Proteomics. Briefly, protein was extracted from LV tissue from control WT and *Isg15^–/–^* mice and WT and *Isg15^–/–^* mice 4 weeks after TAC (*n* = 3 per group). Samples were trypsin-digested, and peptide purification was performed on C18 reversed-phase columns. The peptide mixture was incubated with anti–K-ε-GG antibody–conjugated agarose beads. Nanoscale liquid chromatography–tandem mass spectrometry (nano–LC-MS/MS) analysis was performed using an Ultimate 3000 nano UHPLC system coupled with a Q Exactive HF mass spectrometer (Thermo Fisher Scientific) with an electrospray ionization nanospray source. Raw MS files were analyzed and searched against *Mus musculus* protein database (https://www.uniprot.org/uniprotkb?query=proteome:UP000000589) using MaxQuant (2.0.3.0). The parameters were set as follows: the protein modifications were carbamidomethylation (C), oxidation (M) (variables), and GlyGly (K) (variables); the enzyme was set to trypsin; the maximum missed cleavage was set to 4; the precursor ion mass tolerance was set to 10 ppm; and the MS/MS tolerance was 0.6 Da. Intensity data were logarithmized to obtain a normal or near-normal distribution. Missing values (intensity = 0) were randomly replaced by random values drawn from a similar normal distribution with a smaller mean. Two-way ANOVA and post hoc Tukey’s honestly significant difference test were used to identify significant pairs. Relative quantitation was divided into 2 categories. Difference greater than 0.585 (fold change > 1.5) was considered upregulation, and difference less than –0.585 (fold change < 0.67 [1/1.5]) was considered downregulation. The mass spectrometry proteomics data were deposited to the ProteomeXchange Consortium via the PRIDE (83) partner repository with the data set identifier PXD032267.

### Immunofluorescence microscopy and histology.

Immunofluorescence microscopy for ISG15 and filamin-C was performed with antibodies in the following concentrations: ISG15 (1:100; PA5-88262, Thermo Fisher Scientific), secondary antibody Alexa Fluor 488–AffiniPure Fab fragment donkey anti-rabbit IgG (1:100; 711-547-003, Jackson ImmunoResearch Laboratories Inc.), and Alexa Fluor 648–tagged filamin-C antibody (1:75; NBP2-79816AF647, Novus Biologicals). Slides were visualized with a Zeiss LSM 700 confocal microscope (Carl Zeiss Canada). Paraffin-embedded heart sections were stained with H&E, and myocyte cross-sectional area was determined as previously reported (84, 85) and based on a method adapted from Frustaci and coworkers (86). Myocardial fibrosis was assessed by staining of cardiac cross sections with Picrosirius red. Digitized images were acquired using an AxioScan.Z1 (Carl Zeiss Microscopy). Area of positive (red) staining was determined in a masked manner using HALO.

### Electron microscopy.

Transmission electron microscopy was performed with a Hitachi HT7800 transmission electron microscope (Electron Microscope Research Services, Newcastle University, Newcastle upon Tyne, UK). LV tissue was examined at ×8,000 magnification in approximately 15 fields per mouse and 4–7 mice per group. Mitochondria were counted using ImageJ by an investigator masked to the study groups.

### Untargeted metabolomics.

Untargeted metabolomics was performed at the Segal Cancer Proteomics Centre, Lady Davis Institute for Medical Research, Jewish General Hospital and McGill University (Montreal, Quebec, Canada). Briefly, mouse frozen heart samples were reconstituted in 500 μL of 80% acetonitrile containing 1 μmol/L caffeine-d9 as an internal standard. Samples were homogenized, vortexed, and centrifuged at 20,000*g* for 2 minutes at room temperature. LC-MS analyses were performed with a Vanquish UPLC system (Thermo Fisher Scientific) using a 0%–100% 10-minute gradient from 0.1% formic acid in water to 0.1% formic acid in acetonitrile, at a flow rate of 200 μL/min on a Phenomenex Luna Omega 3 μm Polar C_18_ column, particle size 100 Å, 50 × 2.1 mm. Ten-microliter injection volumes were used for each run. Mass spectra were acquired over the mass range 70–1,000 Da using a Thermo Orbitrap QE+ mass spectrometer operated in the positive and negative ionization modes in separate runs. Data were analyzed using Compound Discover 3.3 (Thermo Fisher Scientific). KEGG pathway analysis was performed using the Functional Analysis tool of MetaboAnalyst 5.0 (87, 88) with input data: *P* value, *m/z*, fold change, and retention time. Pathway enrichment was determined by Fisher’s exact test. Data were deposited with the Metabolomics Workbench (89) and can be accessed by the project DOI: http://dx.doi.org/10.21228/M8WD9H (study ID ST002437).

### Statistics.

Data are expressed as mean ± SD. Statistical significance was determined by 1-way ANOVA with a Tukey’s or Dunnett’s post hoc test as indicated or unpaired 2-tailed Student’s *t* test or unpaired 2-tailed Mann-Whitney test unless otherwise stated. Statistical analyses were performed using GraphPad Prism 9 for macOS (GraphPad Software Inc.). *P* values less than 0.05 were considered statistically significant.

### Study approval.

All animal experimental procedures adhered to the guidelines of the Canadian Council on Animal Care and were approved by the St. Michael’s Hospital Animal Care Committee. For human tissue studies, written informed consent was obtained from patients or substitute decision makers. The study was approved by the Research Ethics Boards of University Health Network (Toronto, Ontario, Canada) and Unity Health Toronto (Toronto, Ontario, Canada) and was conducted in accordance with Declaration of Helsinki principles.

## Author contributions

VGY designed and performed the experiments, analyzed the data, and wrote the paper. SNB designed and performed the experiments, analyzed the data, and revised the paper. HK, MGK, YL, SLA, DTT, SS, and PS performed experiments and analyzed data. FB contributed human data. UK and AOG assisted with proteomics. DOQ, EVP, and CHB performed metabolomics. KAC oversaw cardiac phenotyping. AA designed the experiments, supervised the study, analyzed the data, and wrote the paper.

## Supplementary Material

Supplemental data

## Version 1 05/01/2023

Electronic publication
