# Supplementary material for: Pressure overload induces ISG15 to facilitate adverse ventricular remodeling and promote heart failure
Source: J Clin Invest. 2023 May 1;133(9):e161453. doi: 10.1172/JCI161453 (PMC10145941; doi:10.1172/JCI161453)
Supplement: Supplemental data [file jci-133-161453-s153.pdf]

Supplemental Table 1. Phenotypic characteristics of wildtype and *Ccr2*$^{-/-}$ mice 8 weeks after sham or transverse aortic constriction (TAC) surgery.

	Wildtype sham	Wildtype TAC	*Ccr2*$^{-/-}$ sham	*Ccr2*$^{-/-}$ TAC
n	16	17	14	17
Body weight (g)	28±3	27±3	25±1[a] (not recorded in 1 mouse)	27±2
Heart weight (mg)	132±20	220±55[b]	120±19[c]	185±48[de]
Heart weight:tibial length (mg/mm)	6.4±0.8	10.8±3.0[b]	5.9±0.9[c]	9.2±2.6[de]
Lung weight (mg)	167±26	216±82	164±35	200±76
Systolic blood pressure (mmHg)	102±15	120±39 (not measured in 4 mice)	110±11	114±17

Values are mean ± S.D.. [a]*P* < 0.01 vs. wildtype sham, [b]*P* < 0.0001 vs. wildtype sham, [c]*P* < 0.0001 vs. wildtype TAC, [d]*P* < 0.01 vs. wildtype sham, [e]*P* < 0.001 vs. *Ccr2*$^{-/-}$ sham by one-way ANOVA followed by Tukey's post hoc test.

Supplemental Table 2. Heart rate and chamber dimensions determined by M-mode echocardiography in wildtype and *Ccr2*$^{-/-}$ mice 8 weeks after sham or transverse aortic constriction (TAC) surgery.

	Wildtype sham	Wildtype TAC	*Ccr2*$^{-/-}$ sham	*Ccr2*$^{-/-}$ TAC
n	16	17	14	17
Heart rate (bpm)	385±54	443±55	415±78	459±87[a]
LVDs (mm)	3.2±0.3	3.8±0.7[a]	2.8±0.5[b]	3.1±0.7[c]
LVDd (mm)	4.2±0.2	4.4±0.6	3.9±0.3[c]	4.0±0.5[d]
LVESV (µl)	41±8	65±31[e]	30±11[b]	40±22[c]
LVEDV (µl)	80±10	91±29	65±13[c]	72±25
LVAWT (mm)	0.85±0.12	1.09±0.22[e]	0.88±0.16[d]	1.14±0.22[fg]
LVPWT (mm)	0.76±0.14	1.03±0.25[e]	0.75±0.09[c]	1.12±0.27[hi]

bpm = beats per minute, LVDs = left ventricular internal diameter at systole, LVDd = left ventricular internal diameter at diastole, LVESV = left ventricular end systolic volume, LVEDV = left ventricular end diastolic volume, LVAWT = left ventricular anterior wall thickness, LVPWT = left ventricular posterior wall thickness. Values are mean ± S.D.. [a]$P < 0.05$ vs. wildtype sham, [b]$P < 0.0001$ vs. wildtype TAC, [c]$P < 0.01$ vs. wildtype TAC, [d]$P < 0.05$ vs. wildtype TAC, [e]$P < 0.01$ vs. wildtype sham, [f]$P < 0.001$ vs. wildtype sham, [g]$P < 0.01$ vs. *Ccr2*$^{-/-}$ sham, [h]$P < 0.0001$ vs. wildtype sham, [i]$P < 0.0001$ vs. *Ccr2*$^{-/-}$ sham by one-way ANOVA followed by Tukey's post hoc test.

Supplemental Table 3. Invasive hemodynamic parameters in wildtype and *Ccr2*$^{-/-}$ mice 8 weeks after sham or transverse aortic constriction (TAC) surgery.

	Wildtype sham	Wildtype TAC	*Ccr2*$^{-/-}$ sham	*Ccr2*$^{-/-}$ TAC
n	10	13	13	16
Ejection fraction (%)	52±7	37±10[a]	56±12[b]	45±10[c]
P_{max} (mmHg)	104±13	154±26[d]	101±9[b]	141±22[ef]
ESP (mmHg)	102±14	148±25[d]	97±9[b]	134±22[ef]
EDP (mmHg)	16±9	27±10[g]	16±5[h]	18±8
dP/dt max (mmHg/sec)	5810±1345	5989±1219	6247±1110	6295±1466
dP/dt min (mmHg/sec)	-5284±1463	-6038±1069	-5810±1182	-6172±1706
Tau (ms)	13±4	13±3	11±3	12±3

P_{max} = maximum pressure (peak systolic pressure), ESP = end systolic pressure, EDP = end diastolic pressure. Values are mean ± S.D.. [a]$P < 0.01$ vs. wildtype sham, [b]$P < 0.0001$ vs. wildtype TAC, [c]$P < 0.05$ vs. *Ccr2*$^{-/-}$ sham, [d]$P < 0.0001$ vs. wildtype sham, [e]$P < 0.001$ vs. wildtype sham, [f]$P < 0.0001$ vs. *Ccr2*$^{-/-}$ sham, [g]$P < 0.05$ vs. wildtype sham, [h]$P < 0.05$ vs. wildtype TAC by one-way ANOVA followed by Tukey's post hoc test.

Supplemental Table 4. Top 3 significantly enriched Gene Ontology (GO) results for cardiomyocytes incubated for 24 hours in media conditioned by CCR2+ macrophages isolated from mouse hearts 4 weeks after transverse aortic constriction (TAC).

ID	Term	Count	p_value	FDR
Up differentially expressed genes Biological Process				
GO:0006952	defense_response	99	1.24e-53	4.55e-50
GO:0002376	immune_system_process	121	1.77e-51	3.25e-48
GO:0006955	immune_response	92	6.94e-47	8.48e-44
Up differentially expressed genes Cellular Component				
GO:0005615	extracellular_space	64	8.50e-17	3.27e-14
GO:0005576	extracellular_region	78	9.14e-16	1.76e-13
GO:0005737	cytoplasm	195	5.29e-14	6.79e-12
Up differentially expressed genes Molecular Function				
GO:0042277	peptide_binding	184	3.79e-19	2.22e-16
GO:0033218	amide_binding	184	6.48e-19	2.22e-16
GO:0005515	protein_binding	182	9.54e-19	2.22e-16
Down differentially expressed genes Biological Process				
GO:0030308	negative_regulation_of_cell_growth	3	5.47e-05	2.08e-02
GO:0045926	negative_regulation_of_growth	3	1.61e-04	3.05e-02
GO:0001558	regulation_of_cell_growth	3	5.89e-04	5.60e-02
Down differentially expressed genes Cellular Component				
GO:0005741	mitochondrial_outer_membrane	2	1.85e-03	3.33e-02
GO:0019867	outer_membrane	2	2.25e-03	3.33e-02
GO:0031968	organelle_outer_membrane	2	2.25e-03	3.33e-02
Down differentially expressed genes Molecular Function				
GO:0004842	ubiquitin-protein_transferase_activity	2	1.17e-02	1.13e-01
GO:0019787	ubiquitin-like_protein_transferase_activity	2	1.27e-02	1.13e-01
GO:0016491	oxidoreductase_activity	2	4.60e-02	1.94e-01

Supplemental Table 5. Top 5 significantly enriched KEGG pathways for cardiomyocytes incubated for 24 hours in media conditioned by CCR2+ macrophages isolated from mouse hearts 4 weeks after transverse aortic constriction (TAC).

ID	Term	Count	p_value	FDR
Up differentially expressed genes KEGG pathway				
mmu05168	Herpes_simplex_infection	30	5.09e-17	1.09e-14
mmu04621	NOD-like_receptor_signaling_pathway	25	6.38e-15	6.86e-13
mmu05164	Influenza_A	20	2.56e-10	1.83e-08
mmu05416	Viral_myocarditis	13	2.61e-08	1.40e-06
mmu04612	Antigen_processing_and_presentation	13	3.96e-08	1.49e-06
Down differentially expressed genes KEGG pathway				
None				

Supplemental Table 6. Mouse cytokines and chemokines determined by multiplex assay in media conditioned by CCR2+ macrophages ($CD45^+Ly6c^{hi}CD11b^+CD64^+MHC\text{-}II^{hi}GFP^+$ cells) isolated from mouse hearts 4 weeks after transverse aortic constriction (TAC).

	Median (range)		Median (range)
**No. of cells**	2944 (1195-3343)	**LIF**	Undetectable
**Eotaxin**	Undetectable	**CCL2**	1.07 (0-4.9)
**G-CSF**	Undetectable	**M-CSF**	Undetectable
**GM-CSF**	Undetectable	**CXCL9**	Undetectable
**IFN-γ**	Undetectable	**CCL3**	Undetectable
**IL-1α**	Undetectable	**CCL4**	Undetectable
**IL-1β**	0.75 (0-2.79)	**CCL5**	Undetectable
**IL-2**	Undetectable	**TNFα**	Undetectable
**IL-3**	Undetectable	**VEGF**	Undetectable
**IL-4**	Undetectable	**Erythropoietin**	Undetectable
**IL-5**	Undetectable	**CCL21**	Undetectable
**IL-6**	Undetectable	**CX3CL1**	Undetectable
**IL-7**	1.35 (0.92-2.34)	**IFN-β**	7.82 (0-12.98)
**IL-9**	1.57 (0.56-4.13)	**IL-11**	Undetectable
**IL-10**	Undetectable	**IL-16**	1.98 (0.8-2.52)
**IL-12 (p40)**	Undetectable	**IL-20**	Undetectable
**IL-12 (p70)**	Undetectable	**CCL22**	Undetectable
**IL-13**	Undetectable	**CCL12**	0.57 (0.30-1.07)
**IL-15**	5.61 (5.12-9.00)	**CCL20**	Undetectable
**IL-17**	Undetectable	**CCL19**	Undetectable
**IP-10**	0.68 (0.42-0.78)	**CCL17**	Undetectable
**CXCL1**	Undetectable	**TIMP-1**	Undetectable

Values are in pg/mL. Analytes were measured using the Eve Technologies Mouse Cytokine 44-Plex Discovery Assay. Values for LIX (CXCL5) are not presented because the assay reports that LIX results are not validated. Values are reported as undetectable if the median concentration fell below the 4 or 5 parameter logistic standard curve.

Supplemental Table 7. Summary of the number of differentially regulated diGLY sites in left ventricles of wildtype and *Isg15*$^{-/-}$ mice 4 weeks after transverse aortic constriction (TAC).

	Upregulated (fold change >1.5)	Downregulated (fold change <1/1.5)
Wildtype TAC vs. wildtype control	10	48
Isg15$^{-/-}$ TAC vs. *Isg15*$^{-/-}$ control	74	18
Isg15$^{-/-}$ TAC vs. wildtype TAC	31	5

Supplemental Table 8. Significantly upregulated diGLY sites in left ventricles of wildtype mice 4 weeks after transverse aortic constriction compared to control wildtype mice.

Protein name	Position	GlyGly (K) probabilities	p_value	Difference
[Protein ADP-ribosylarginine] hydrolase-like protein 1	335	GLYQELEHK(1)GR	0.038957	1.319833
Xin actin-binding repeat-containing protein 2	1054	GISAQEIQAGNVK(1)SAR	0.028129	1.823933
Titin	12993	TSTAK(1)LIVEELPVR	0.007273	5.088633
Synaptopodin 2-like protein	433	NSPNPELLSLVQNLDEK(1)PR	0.003149	1.3043
Histone-lysine N-methyltransferase 2B	102	VQLFK(0.994)IDQQQQQK(0.006)	0.020353	2.701133
Atypical chemokine receptor 3	362	VSETEYSALEQNTK(1)	0.049858	0.850433
Filamin-C	2590	YGGPQHIVGSPFK(0.997)AK(0.003)	0.002609	2.131467
Myosin-7	1279	AK(1)LQTENGELSR	0.025301	3.147833
Myosin-7	1305	GK(1)LTYTQQLEDLKR	0.017522	3.780733
Myosin-7	34	LEAQTRPFDLK(1)K	0.009401	3.913867

Supplemental Table 9. Phenotypic characteristics of wildtype and *Isg15*$^{-/-}$ mice 8 weeks after sham or transverse aortic constriction (TAC).

	Wildtype sham	Wildtype TAC	*Isg15*$^{-/-}$ sham	*Isg15*$^{-/-}$ TAC
n	15	13	13	14
Body weight (g)	29±4	28±2	28±2	28±1
Heart weight (mg)	114±13	190±40[a]	122±16[b]	171±38[ac]
Heart weight:tibial length (mg/mm)	5.9±0.6	10.1±2.2[a]	6.2±0.7[b]	8.8±19[ac]
Lung weight (mg)	180±20	227±114	169±20	217±101
Systolic blood pressure (mmHg)	100±11	103±17 (not measured in 2 mice)	110±8	113±10[d]

Values are mean ± S.D.. [a]*P* < 0.0001 vs. wildtype sham, [b]*P* < 0.0001 vs. wildtype TAC, [c]*P* < 0.001 vs. *Isg15*$^{-/-}$ sham, [d]*P* < 0.05 vs. wildtype sham by one-way ANOVA followed by Tukey's post hoc test.

Supplemental Table 10. Heart rate and chamber dimensions determined by M-mode echocardiography in wildtype and *Isg15*$^{-/-}$ mice 8 weeks after sham or transverse aortic constriction (TAC).

	Wildtype sham	Wildtype TAC	*Isg15*$^{-/-}$ sham	*Isg15*$^{-/-}$ TAC
n	15	12	13	14
Heart rate (bpm)	363±56	384±66	399±38	384±76
LVDs (mm)	3.1±0.6	3.6±0.7	3.2±0.6	2.9±0.9[a]
LVDd (mm)	4.1±0.3	4.2±0.6	4.1±0.4	4.1±0.6
LVESV (µl)	40±15	58±22	43±5	43±24
LVEDV (µl)	76±13	81±23	75±19	76±24
LVAWT (mm)	0.84±0.11	1.09±0.23[b]	0.87±0.13[a]	1.12±0.18[cd]
LVPWT (mm)	0.76±0.11	1.00±0.05[c]	0.65±0.07[e]	0.92±0.17[fg]

bpm = beats per minute, LVDs = left ventricular internal diameter at systole, LVDd = left ventricular internal diameter at diastole, LVESV = left ventricular end systolic volume, LVEDV = left ventricular end diastolic volume, LVAWT = left ventricular anterior wall thickness, LVPWT = left ventricular posterior wall thickness. Values are mean ± S.D.. [a]$P < 0.05$ vs. wildtype TAC, [b]$P < 0.01$ vs. wildtype sham, [c]$P < 0.001$ vs. wildtype sham, [d]$P < 0.01$ vs. *Isg15*$^{-/-}$ sham, [e]$P < 0.0001$ vs. wildtype TAC, [f]$P < 0.05$ vs. wildtype sham, [g]$P < 0.0001$ vs. *Isg15*$^{-/-}$ sham by one-way ANOVA followed by Tukey's post hoc test.

Supplemental Table 11. Invasive hemodynamic parameters in wildtype and *Isg15*$^{-/-}$ mice 8 weeks after sham or transverse aortic constriction (TAC).

	Wildtype sham	Wildtype TAC	*Isg15*$^{-/-}$ sham	*Isg15*$^{-/-}$ TAC
n	15	10	13	13
Ejection fraction (%)	59±19	39±23	57±17	53±23
P_{max} (mmHg)	100±17	143±30[a]	101±12[b]	162±18[ac]
ESP (mmHg)	96±17	136±25[a]	95±13[b]	147±17[ac]
EDP (mmHg)	14±5	15±6	14±5	18±7
dP/dt max (mmHg/sec)	6085±1751	6935±3363	6269±2206	8063±1884
dP/dt min (mmHg/sec)	-5205±1606	-6624±2760	-5963±2048	-7406±2120
Tau (ms)	13±4	13±8	11±2	10±3

P_{max} = maximum pressure (peak systolic pressure), ESP = end systolic pressure, EDP = end diastolic pressure. Values are mean ± S.D.. [a]P < 0.0001 vs. wildtype sham, [b]P < 0.0001 vs. wildtype TAC, [c]P < 0.0001 vs. *Isg15*$^{-/-}$ sham by one-way ANOVA followed by Tukey's post hoc test.

Supplemental Table 12. Significantly enriched KEGG pathways in the untargeted metabolomic comparison of *Isg15*$^{-/-}$ and wildtype mouse hearts 8 weeks after transverse aortic constriction (TAC).

Pathway	Pathway total	Hits.total	Hits.significant	Expected	p_value
D-Glutamine and D-glutamate metabolism	6	6	6	0.5343	0.000275
β-Alanine	21	8	5	1.0686	0.001367
Glutathione metabolism	19	6	4	0.8014	0.003368
Porphyrin and chlorophyll metabolism	27	5	4	0.5343	0.008044
Arginine and proline metabolism	37	23	12	2.6715	0.009076
Alanine, apartate and glutamate metabolism	28	12	6	1.6029	0.012798
Arginine biosynthesis	14	8	4	1.0686	0.012836
Histidine metabolism	16	7	4	1.0686	0.012836
Aminoacyl-tRNA biosynthesis	22	18	7	2.9386	0.016215
Nitrogen metabolism	6	2	2	0.26715	0.017423
Lysine degradation	19	8	4	1.2022	0.020886
Pantothenate and CoA biosynthesis	17	8	4	1.4693	0.044731

Supplemental Table 13. Antibodies used for flow cytometric identification of CCR2+ cardiac macrophages.

Surface Marker	Fluorescence Tag	Clone	Catalogue No.	Source
Ly6c	BV 605	HK 1.4	128035	Biolegend
CD45	PE/Cy7	30-F11	103114	Biolegend
CD11b	PE	M1/70	101207	Biolegend
CD64	PE/Dazzle 594	X54-5/7.1	139319	Biolegend
MHC-II	Alexa Fluor 700	M5/114.15.2	56-5321-80	eBioscience

Supplemental Table 14. Primer sequences used in quantitative reverse transcription polymerase chain reaction (qRT-PCR) experiments.

Species	Gene name	Forward primer sequence (5'->3')	Reverse primer sequence (5'->3')
Mus musculus	*Rpl13a*	GCTCTCAAGGTTGTTCGGCTGA	AGATCTGCTTCTTCTTCCGATA
Mus musculus	*Irf7*	CAGCGAGTGCTGTTTGGAGA	AAGTTCGTACACCTTATGCGG
Mus musculus	*Isg15*	TGGTACAGAACTGCAGCGAG	AGCCAGAACTGGTCTTCGTG
Mus musculus	*Bst2*	ACATGGCGCCCTCTTTCTATCACT	TGACGGCGAAGTAGATTGTCAGGA
Mus musculus	*Ifit1*	CTGAGATGTCACTTCACATGGAA	GTGCATCCCCAATGGGTTCT
Mus musculus	*Ifit3*	CCTACATAAAGCACCTAGATGGC	ATGTGATAGTAGATCCAGGCGT
Mus musculus	*Gvin1*	GAGAGACTGCAAGGAAGCCAAAG	GGTGCCAAAGTTGTCCTTGAAGG
Mus musculus	*Oasl2*	GATGGATATCCTCCCAGCTTACG	TTGGTGAGAAGTCACCAGGGTAG
Mus musculus	*Lgals3bp*	TGGAACCTTTTGGATGCCCA	GAAGCCCCGTGGTATCGTT
Mus musculus	*Ifitm3*	CCCCCAAACTACGAAAGAATCA	ACCATCTTCCGATCCCTAGAC
Mus musculus	*Ifi2712a*	CTGTTTGGCTCTGCCATAGGAG	CCTAGGATGGCATTTGTTGATGTGG
Homo sapiens	*ISG15*	GCGCAGATCACCCAGAAGAT	GTTCGTCGCATTTGTCCACC
Homo sapiens	*GATA4*	ACCCCAATCTCGTAGATATGTTT	AGGCGTTGCACAGATAGTGA
Homo sapiens	*ACTC1*	TGTGCCAAGATGTGTGACGA	AGGGTCAGGATGCCTCTCTT
Homo sapiens	*RPL13A*	TCGTACGCTGTGAAGGCATC	TTTTGTGGGGCAGCATACCT
Homo sapiens	*RPS18*	TGATCCCTGAAAAGTTCCAGCA	CTTCGGCCCACACCCTTAAT

SUPPLEMENTAL FIGURE LEGENDS

Supplemental Figure 1. Multi-color flow cytometry gating strategy and enumeration of CCR2+ cardiac monocyte-derived macrophages ($CD45^+Ly6c^{hi}CD11b^+CD64^+MHC\text{-}II^{hi}GFP^+$ cells).

Supplemental Figure 2. Flow diagram showing outcomes for wildtype and *Ccr2*$^{-/-}$ mice subjected to sham or transverse aortic constriction (TAC).

Supplemental Figure 3. Representative M-mode echocardiographs (A) and (B) fractional shortening, (C) cardiac output and (D) stroke volume in wildtype and *Ccr2*$^{-/-}$ mice 8 weeks after sham or TAC surgery. Wildtype sham (n=16), wildtype TAC (n=17), *Ccr2*$^{-/-}$ sham (n=14), *Ccr2*$^{-/-}$ TAC (n=17). Values are mean ± S.D.. *$P < 0.05$, **$P < 0.01$, ***$P < 0.001$, ****$P < 0.0001$ by one-way ANOVA followed by Tukey's post hoc test.

Supplemental Figure 4. (A) Heat map showing genes differentially expressed in adult mouse cardiomyocytes incubated in control media or media conditioned by CCR2+ cardiac macrophages for 24 hours, fold change ≥ 2.5 fold, $P < 0.05$. (B) Venn diagram of genes differentially regulated in adult mouse cardiomyocytes incubated for 24 hours in media conditioned by CCR2+ macrophages ($CD45^+Ly6c^{hi}CD11b^+CD64^+MHC\text{-}II^{hi}GFP^+$ cells) isolated from mouse hearts 4 weeks after transverse aortic constriction (TAC). The gene symbols for the 42 genes observed to be (≥ 2.5 fold) differentially expressed by RNA sequencing (A) were entered into Interferome v.2.01 and the database was searched for previous entries where genes have been up- or down-regulated ≥ 2 fold (default settings). Note, 39/42 of the differentially expressed genes have previously been observed to be regulated by either type I or type II interferons. Because there are more datasets available for genes regulated by type I interferons than types II or III, caution should be used in interpreting low or negative results for type II or type III interferons.

Supplemental Figure 5. Quantitative reverse transcription polymerase chain reaction (qRT-PCR) for interferon response genes (*Isg15*, *Irf7*, *Ifit1*, *Ifit3*, *Ifi2712a*, *Ifitm3*, *Oasl2*, *Lgals3bp*, *Bst2*, *Gvin1*) in mouse hearts 1, 4 or 8 weeks after TAC in comparison to control hearts 8 weeks after sham surgery (n=5/group). Values are mean ± S.D.. *P < 0.05, **P < 0.01 by one-way ANOVA followed by Dunnett's post hoc test.

Supplemental Figure 6. Flow cytometry determination of CCR2 expression in CD45+CD64+CD11b+ bone marrow-derived macrophages (BMDMs) from wildtype and *Ccr2*$^{gfp/+}$ mice, demonstrating that BMDMs are CCR2 positive.

Supplemental Figure 7. Flow cytometry histograms of CD4+ T cells isolated from mouse spleens (left unstained; right anti-CD4 stained), confirming >97% purity of the cell population for CD4+ cells.

Supplemental Figure 8. Immunoblotting mouse hearts 1, 4 or 8 weeks after TAC, in comparison to control hearts 8 weeks after sham surgery for (A) cGAS, (B) STING, (C) RIG-I and (D) MAVS (n=5/group). Values are mean ± S.D.. *P < 0.05, **P < 0.01 by one-way ANOVA followed by Dunnett's post hoc test.

Supplemental Figure 9. (D) Immunoblotting for ISG15 in mouse cardiomyocytes following stimulation with 500IU/mL IFN-α or 500ng/mL poly(I:C) LMW/LyoVec for 48 hours (n=5/condition). Values are mean ± S.D.. ****P < 0.0001 by one-way ANOVA followed by Dunnett's post hoc test.

Supplemental Figure 10. Phase contrast image showing human cardiac myocytes after culture for 21 days, with their elongated appearance, beginning to form myotube-like structures. Scale bar = 200µm.

Supplemental Figure 11. Echocardiographic parameters of wildtype mice 4 weeks after sham (n=16) or transverse aortic constriction (TAC; n=17). (A) Left ventricular mass. (B) Ejection fraction. (C) Fractional shortening. (D) Cardiac output. (E) Stroke volume. $^{**}P < 0.01$, $^{****}P < 0.0001$ by unpaired two-tailed Student *t* test.

Supplemental Figure 12. Dual immunofluorescence staining for ISG15 and filamin-C in the hearts of sham-operated *Isg15*$^{-/-}$ mice and *Isg15*$^{-/-}$ mice 1 week after TAC. Scale bar = 10μm.

Supplemental Figure 13. Flow diagram showing outcomes for wildtype and *Isg15*$^{-/-}$ mice subjected to sham or transverse aortic constriction (TAC).

Supplemental Figure 14. Cardiomyocyte size, interstitial fibrosis and mitochondrial density in wildtype and *Isg15*$^{-/-}$ mice 8 weeks after sham or transverse aortic constriction (TAC). (A) H&E stained cardiac cross sections and myocyte cross sectional area. Scale bar = 50μm. (B) Picrosirius red staining and quantitation of cardiac picrosirius red positive area. Scale bar = 100μm. Wildtype sham (n=15), wildtype TAC (n=12), *Isg15*$^{-/-}$ sham (n=13), *Isg15*$^{-/-}$ TAC (n=14) for each, except (B) wildtype sham (n=14). (C) Representative transmission electron micrographs of cardiomyocytes from wildtype and *Isg15*$^{-/-}$ mice 8 weeks after sham or TAC surgery and quantitation of mitochondria number per x8000 field in approximately 15 fields per mouse. Scale bar = 1μm. Wildtype sham (n=4), wildtype TAC (n=5), *Isg15*$^{-/-}$ sham (n=6), *Isg15*$^{-/-}$ TAC (n=7). Values are mean ± S.D. $^{*}P < 0.05$, $^{**}P < 0.01$, $^{***}P < 0.001$, $^{****}P < 0.0001$ by one-way ANOVA followed by Tukey's post hoc test (skew distributed data in B were log-transformed prior to statistical comparison).

Supplemental Figure 15. RNAscope in situ hybridization for *Ccr2* and immunofluorescence staining for Troponin I in heart sections of *Isg15*$^{-/-}$ mice 8 weeks after sham surgery and *Isg15*$^{-/-}$ mice 1, 4 and 8 weeks after transverse aortic constriction (TAC). Scale bar = 10μm. Quantitation of *Ccr2* expressing cells per cardiac section. *Isg15*$^{-/-}$ sham (n=6), *Isg15*$^{-/-}$ 1 week TAC (n=5), *Isg15*$^{-/-}$ 4 weeks TAC (n=4),

Isg15$^{-/-}$ 8 weeks TAC (n=5)). Values are mean ± S.D.. ****P < 0.0001 by one-way ANOVA followed by Dunnett's post hoc test.

Supplemental Figure 16. Principal component analysis (PCA) plots following untargeted metabolomics in hearts of wildtype (WT) and *Isg15*$^{-/-}$ (KO) mice 8 weeks after sham or TAC surgery. Wildtype sham (n=3), wildtype TAC (n=4), *Isg15*$^{-/-}$ sham (n=4), *Isg15*$^{-/-}$ TAC (n=4).

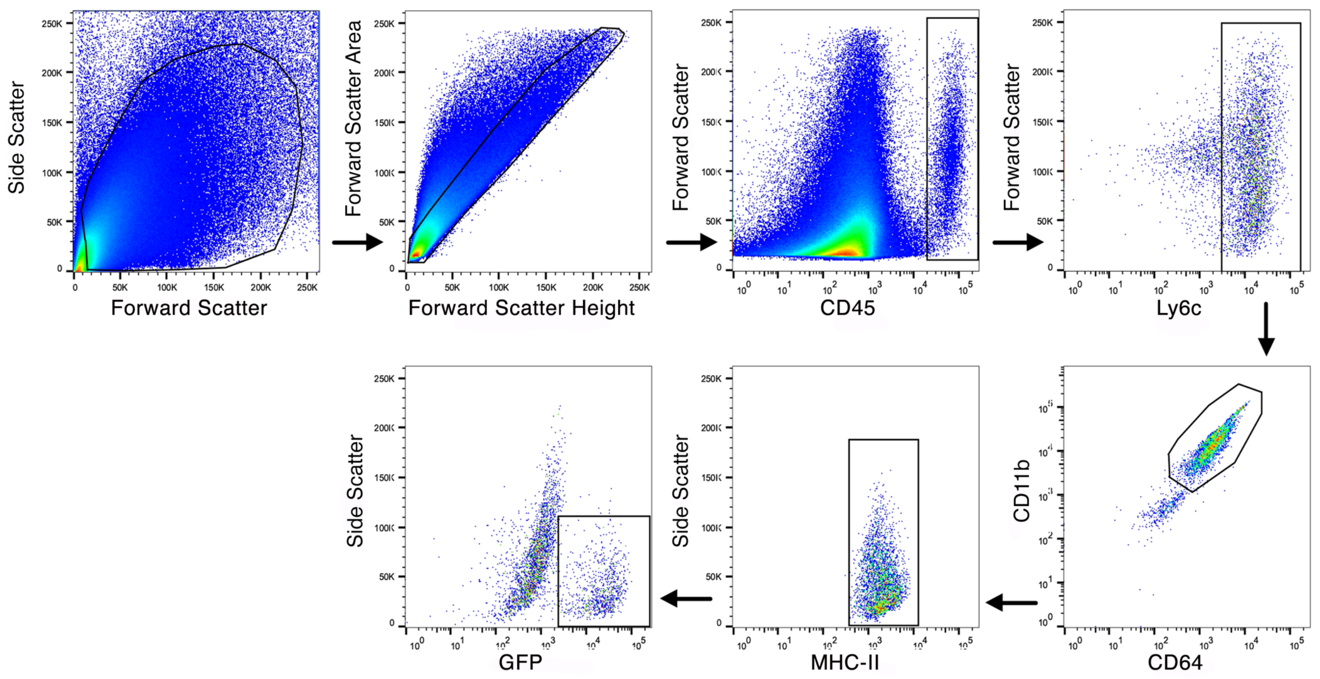

Supplemental Figure 1.

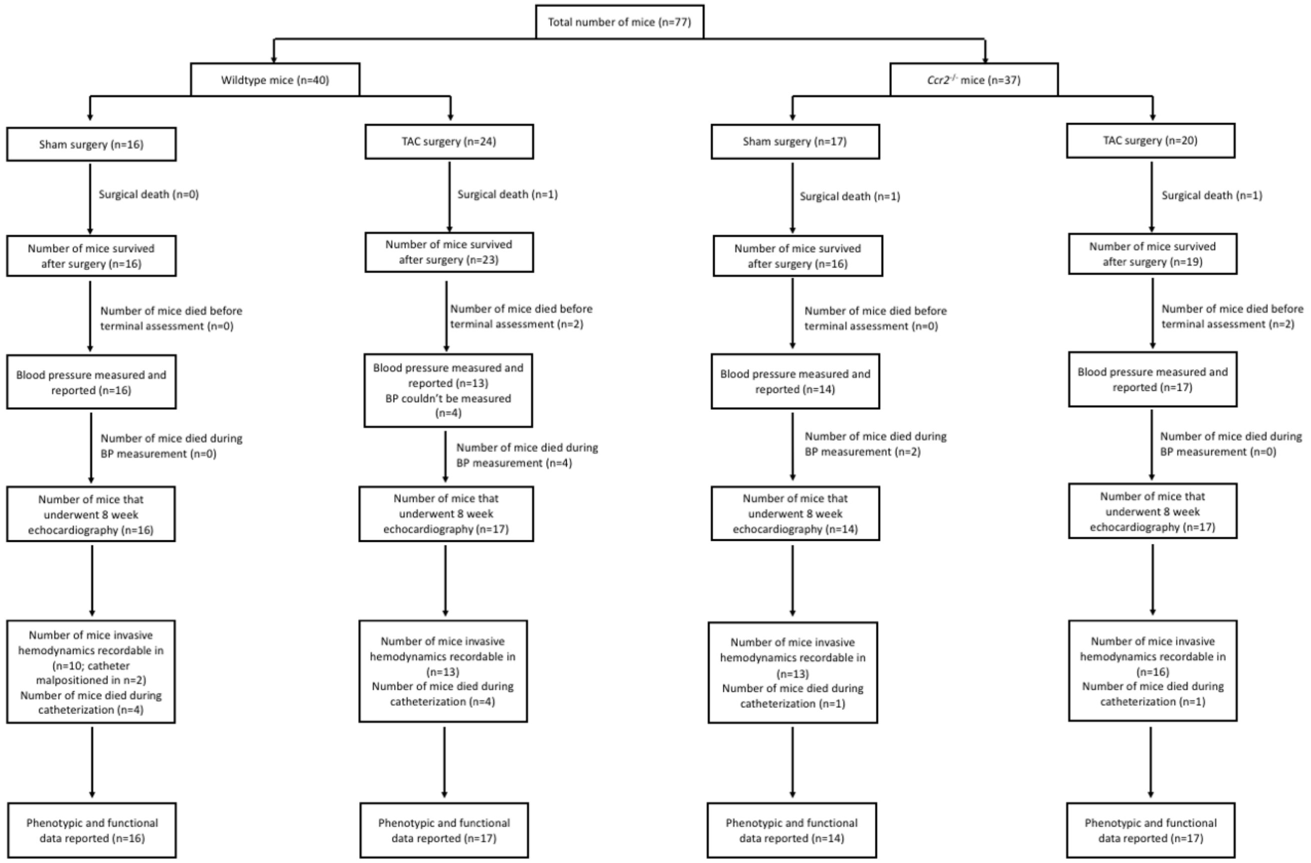

Supplemental Figure 2.

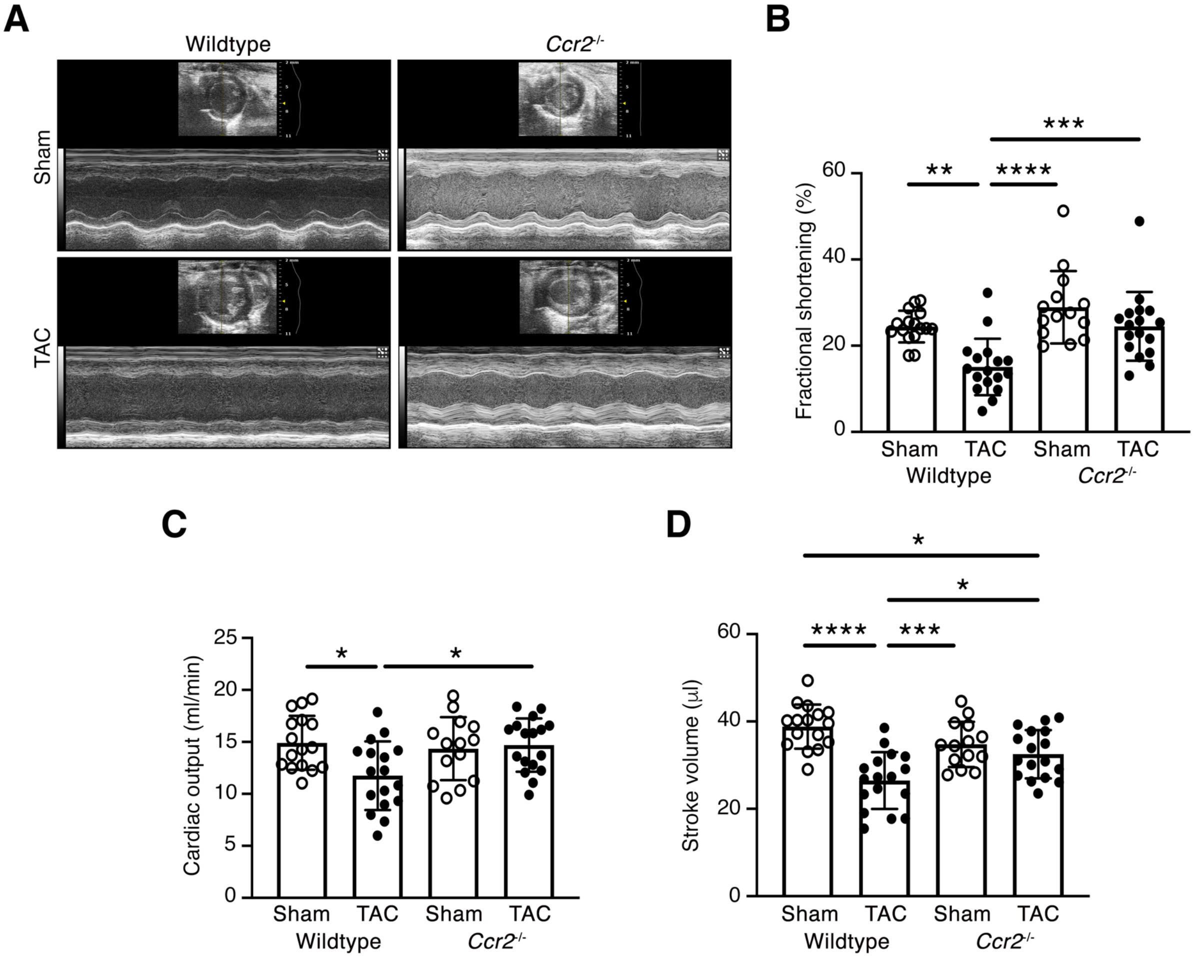

Supplemental Figure 3.

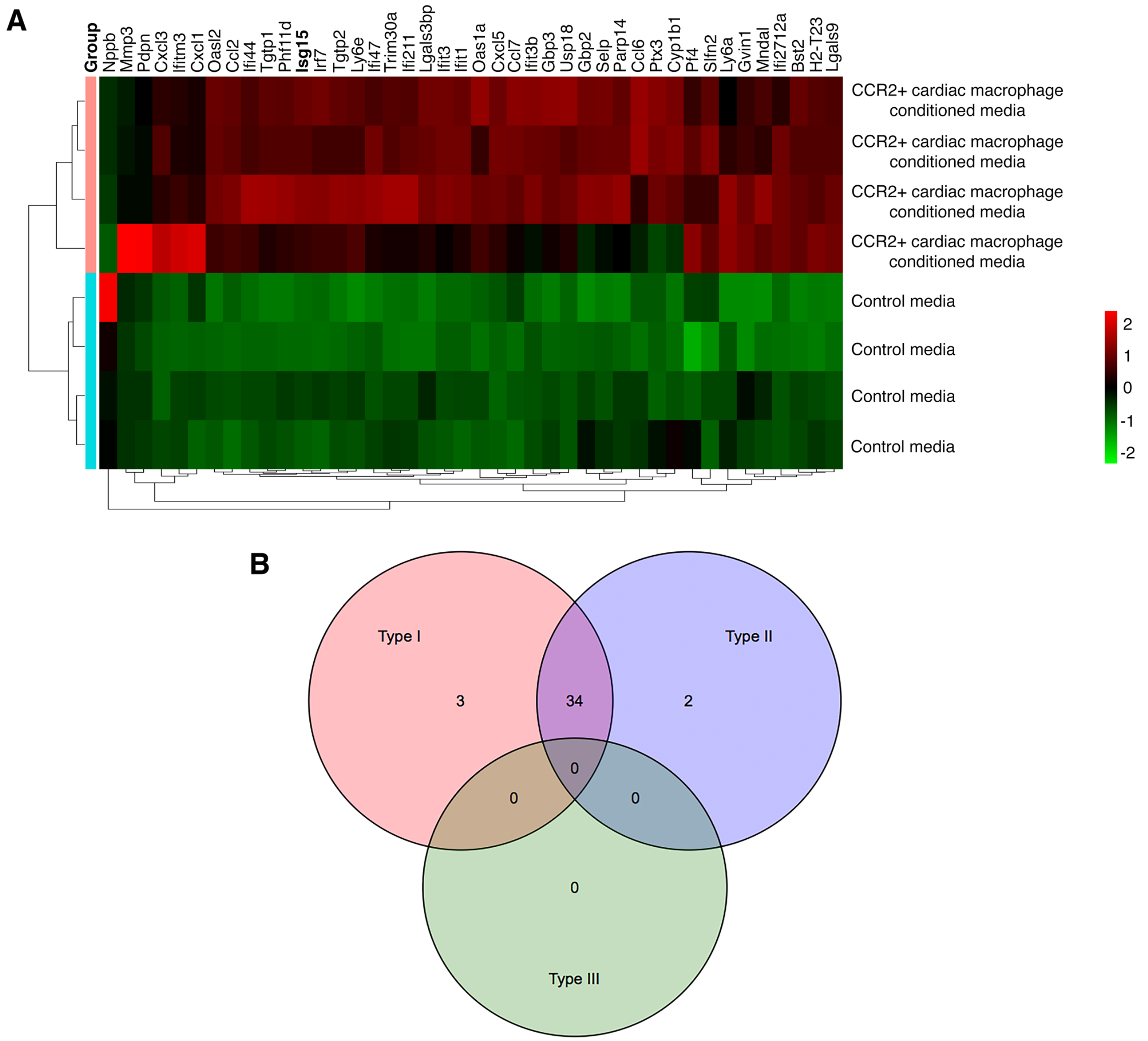

Supplemental Figure 4.

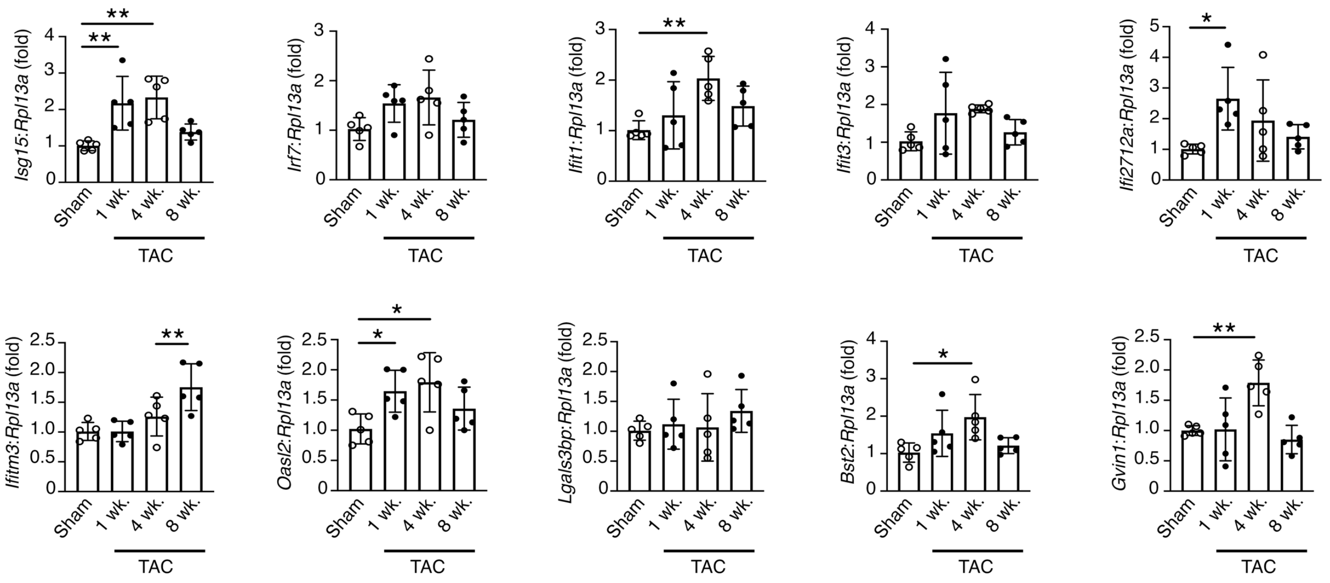

Supplemental Figure 5.

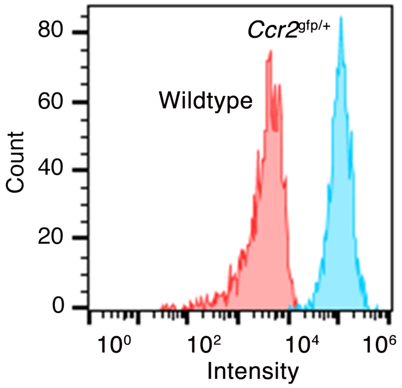

Supplemental Figure 6.

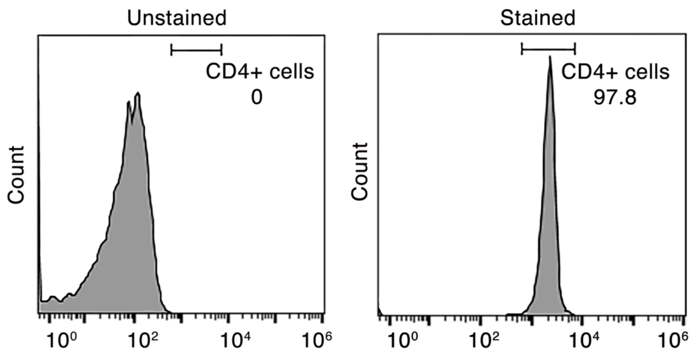

Supplemental Figure 7.

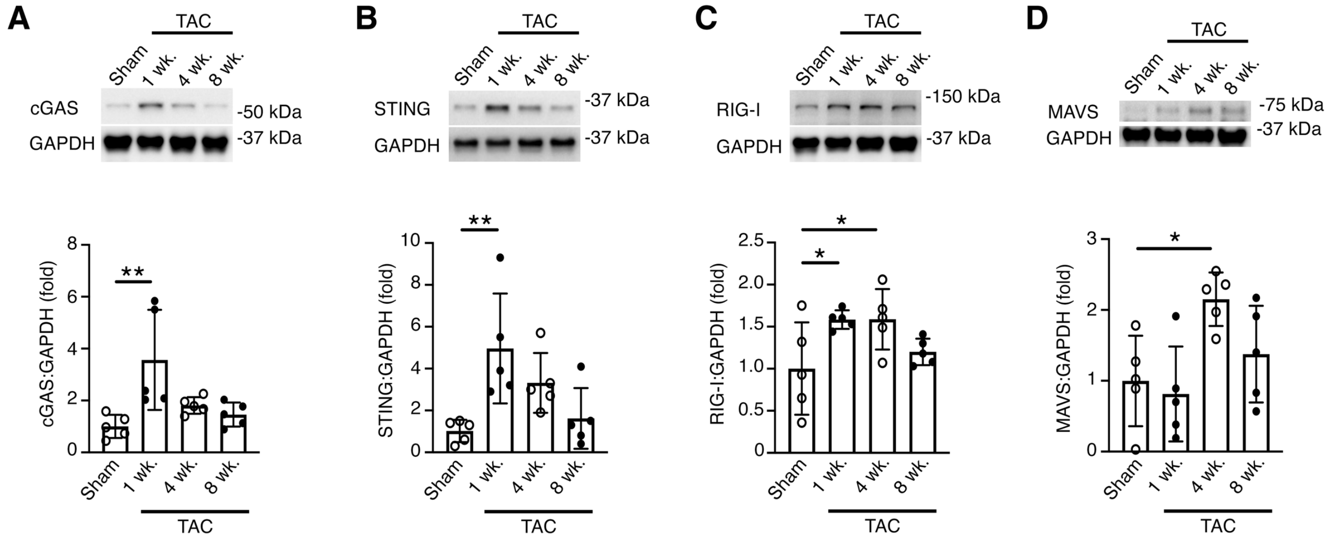

Supplemental Figure 8.

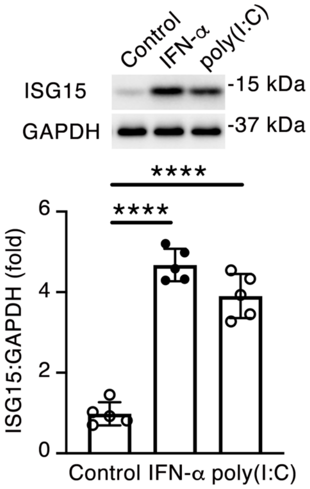

Supplemental Figure 9.

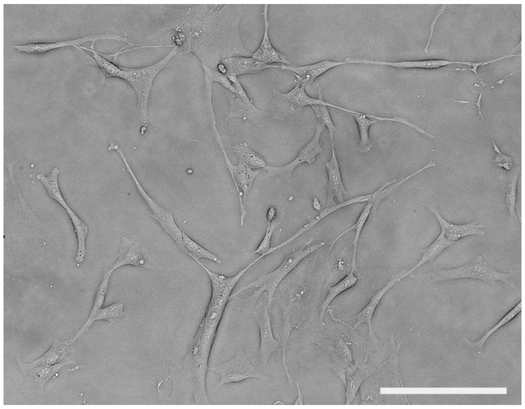

Supplemental Figure 10.

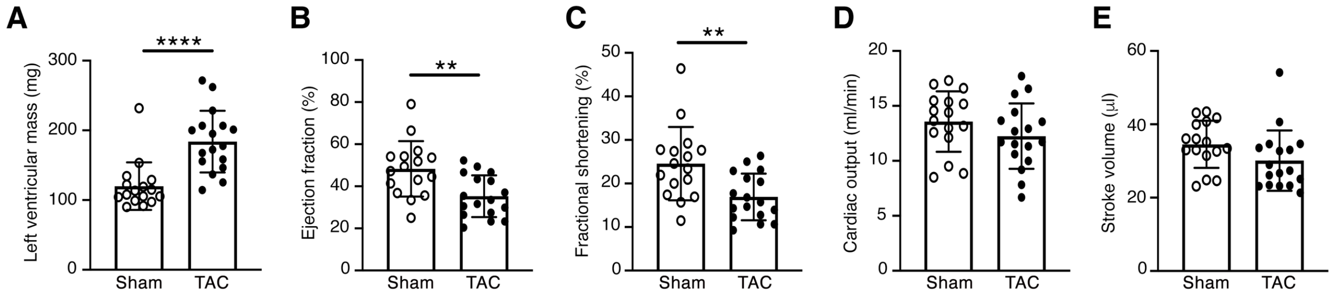

Supplemental Figure 11.

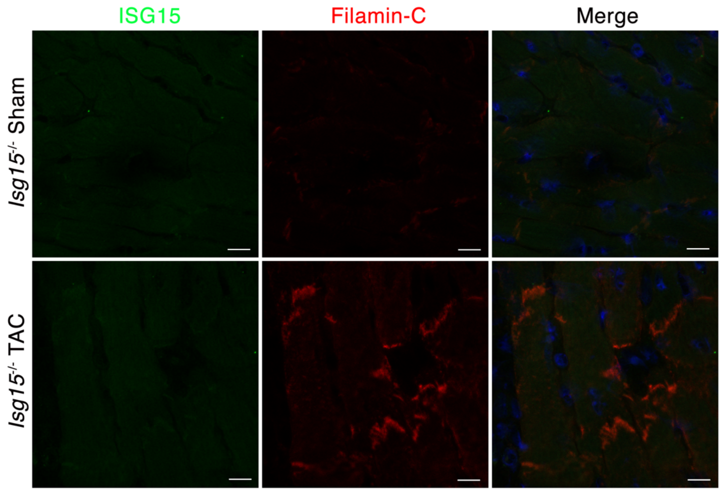

Supplemental Figure 12.

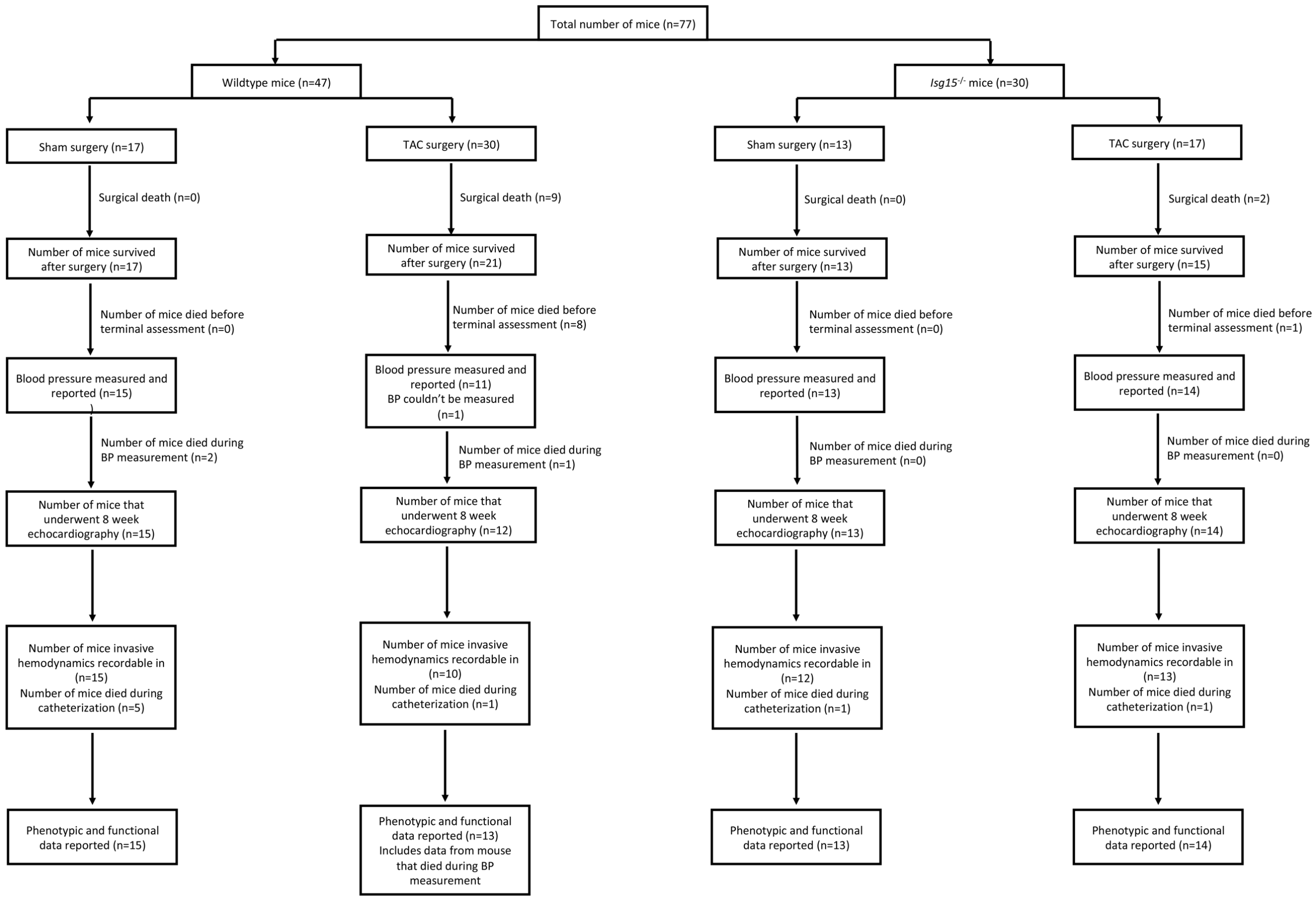

Supplemental Figure 13.

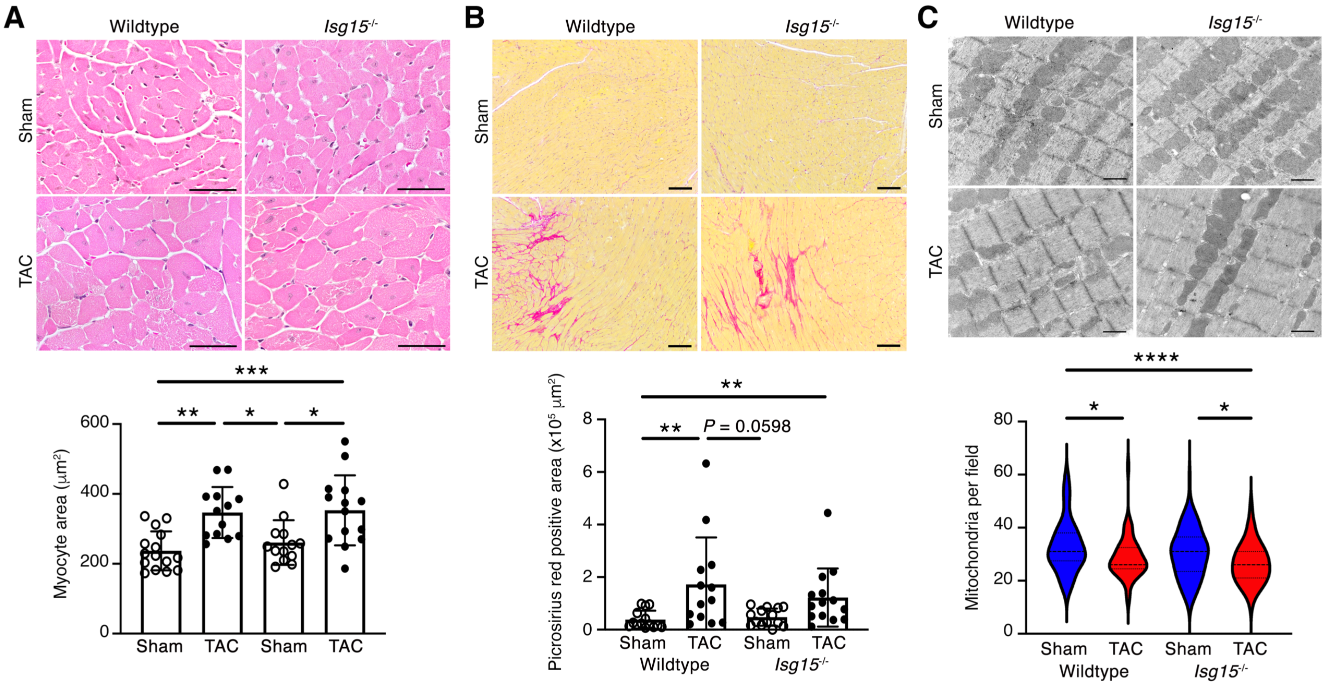

Supplemental Figure 14.

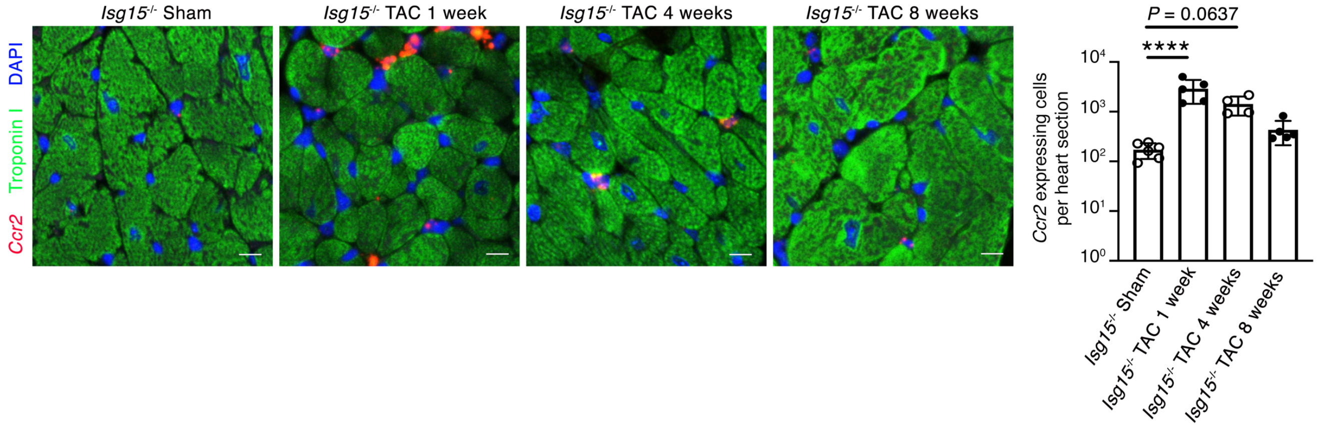

Supplemental Figure 15.

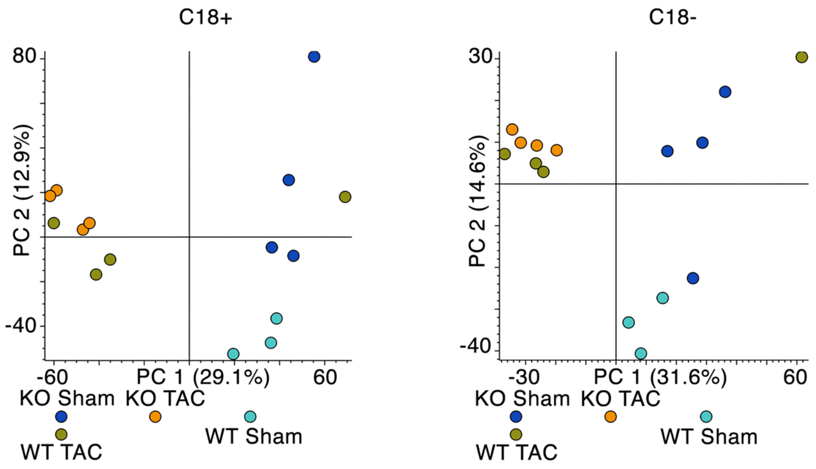

Supplemental Figure 16.
